# Anatomical Variations in the Sinoatrial Nodal Artery: A Meta-Analysis and Clinical Considerations

**DOI:** 10.1371/journal.pone.0148331

**Published:** 2016-02-05

**Authors:** Jens Vikse, Brandon Michael Henry, Joyeeta Roy, Piravin Kumar Ramakrishnan, Wan Chin Hsieh, Jerzy A. Walocha, Krzysztof A. Tomaszewski

**Affiliations:** 1 International Evidence-Based Anatomy Working Group, Krakow, Poland; 2 Department of Anatomy, Jagiellonian University Medical College, Krakow, Poland; 3 First Faculty of Medicine, Charles University, Prague, Czech Republic; University of Palermo, ITALY

## Abstract

**Background and Objective:**

The sinoatrial nodal artery (SANa) is a highly variable vessel which supplies blood to the sinoatrial node (SAN). Due to its variability and susceptibility to iatrogenic injury, our study aimed to assess the anatomy of the SANa and determine the prevalence of its anatomical variations.

**Study Design:**

An extensive search of major electronic databases was performed to identify all articles reporting anatomical data on the SANa. No lower date limit or language restrictions were applied. Anatomical data regarding the artery were extracted and pooled into a meta-analysis.

**Results:**

Sixty-six studies (n = 21455 hearts) were included in the meta-analysis. The SANa usually arose as a single vessel with a pooled prevalence of 95.5% (95%CI:93.6–96.9). Duplication and triplication of the artery were also observed with pooled prevalence of 4.3% (95%CI:2.8–6.0) and 0.3% (95%CI:0–0.7), respectively. The most common origin of the SANa was from the right coronary artery (RCA), found in 68.0% (95%CI:55.6–68.9) of cases, followed by origin from the left circumflex artery, and origin from the left coronary artery with pooled prevalence of 22.1% (95%CI:15.0–26.2) and 2.7 (95%CI:0.7–5.2), respectively. A retrocaval course of the SANa was the most common course of the artery with a pooled prevalence of 47.1% (95%CI:36.0–55.5). The pooled prevalence of an S-shaped SANa was 7.6% (95%CI:2.9–14.1).

**Conclusions:**

The SANa is most commonly reported as a single vessel, originating from the RCA, and taking a retrocaval course to reach the SAN. Knowledge of high risk anatomical variants of the SANa, such as an S-shaped artery, must be taken into account by surgeons to prevent iatrogenic injuries. Specifically, interventional or cardiosurgical procedures, such as the Cox maze procedure for atrial fibrillation, open heart surgeries through the right atrium or intraoperative cross-clamping or dissection procedures during mitral valve surgery using the septal approach can all potentiate the risk for injury in the setting of high-risk morphological variants of the SANa.

## Introduction

The sinoatrial nodal artery (SANa) is a branch of the coronary circulation, that supplies blood to the sinoatrial node (SAN), Bachmann's bundle, crista terminalis, and the left and right atrial free walls[1]. Due to the highly variable anatomical characteristics of the artery, a consensus has yet to be reached on its normal anatomy. Furthermore, detailed anatomical knowledge of the artery and its variations is essential to understand both cardiac disease processes and avoiding iatrogenic injuries during interventional cardiology or cardiosurgical procedures[2,3].

**Fig 1:**
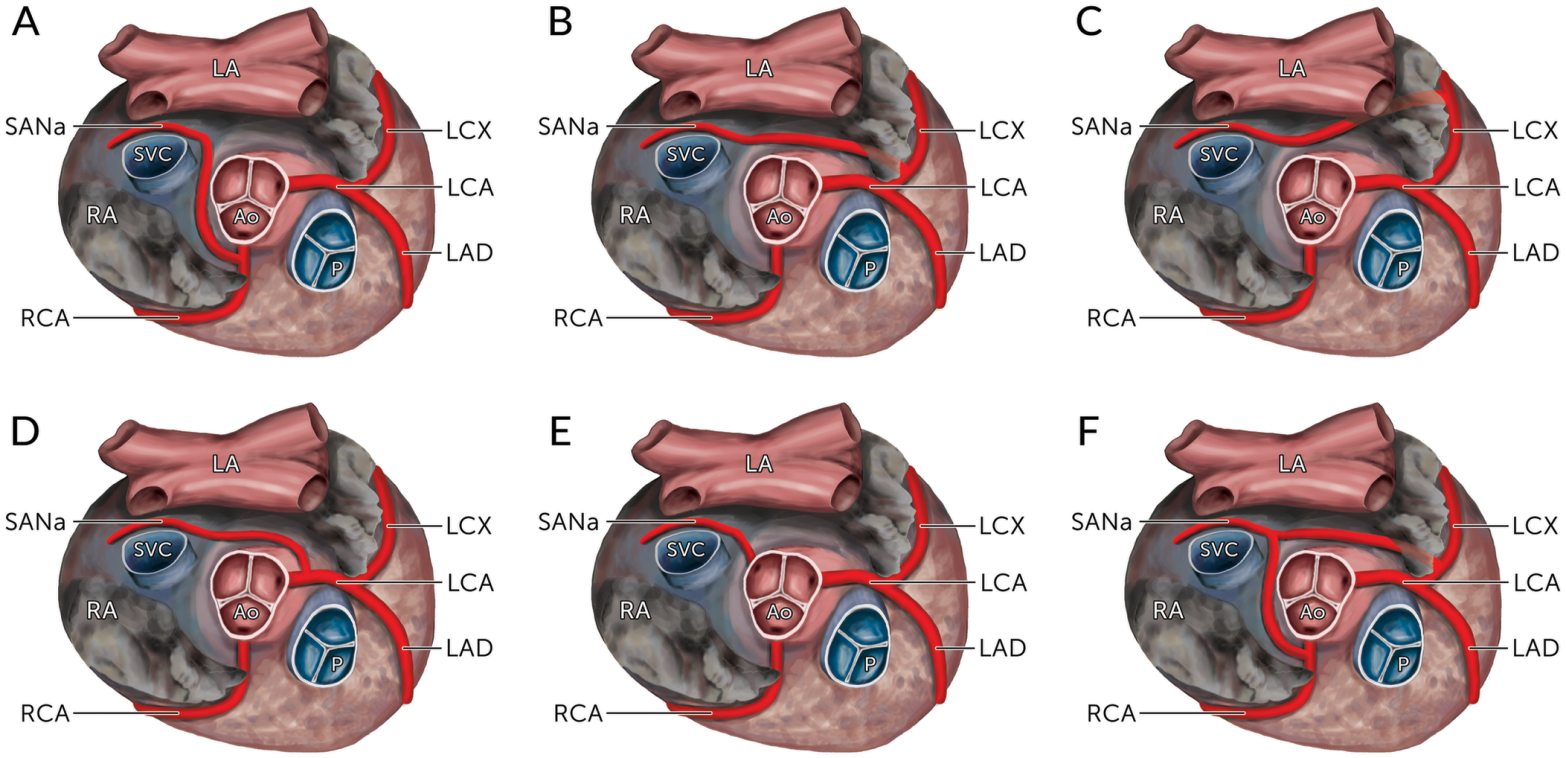
The various origins of the sinoatrial nodal artery (SANa) as seen from a superior view with their calculated pooled prevalence in the overall population. (A) From the Right Coronary Artery; (B) From the Left Circumflex Artery (proximal); (C) From the Left Circumflex Artery (distal); (D) From the Left Coronary Artery; (E) From the Aorta; (F) Dual origin from the Right Coronary Artery and the Left Circumflex Artery. The prevalence rates for B and C are reported as a combined rate. LA, Left Atrium; RA, Right Atrium; SVC, Superior Vena Cava; Ao, Aorta; P, Pulmonary Trunk; RCA, Right Coronary Artery; LCA, Left Coronary Artery; LCX, Left Circumflex Artery; LAD, Left Anterior Descending Artery; SANa, Sinoatrial Nodal Artery.

Previous reports in literature have stated that the SANa most frequently originates from either the right coronary artery (RCA) or the left circumflex branch (LCX) of the left coronary artery (LCA)[4]. When originating from the RCA (Fig 1A), the SANa tends to arise from the proximal segment, contrary to its origin from the LCX, which can be proximal (Fig 1B) or distal (Fig 1C). Furthermore, origins of the artery directly from the LCA have also been frequently reported[5–22] (Fig 1D). In rare cases, extra-coronary origins of SANa, including from the aorta[9,12,23–26] (Fig 1E) and the bronchial artery[10,26,27], have been observed as well.

Although only a single SANa is usually present in a human heart, both duplication (Fig 1F), and more rarely triplication of the artery, have also been reported. Since the artery exists most commonly as a single branch without collateral circulation[3,7,8,10–13,15–59], the SAN, supplied by the SANa, is susceptible to ischemic damage during vasoocclusive disease of the vessel. However, one study[37] investigating 106 hearts from Japanese cadavers found that 53.8% of the studied hearts had two or more SANa, which may indicate an anatomical heterogeneity in the SAN circulation among different populations.

The micro-vascularization of the SAN differs from other atrial tissue by consisting of a highly dense plexus of arterioles and capillaries[11,60]. This rich vascularization facilitates adequate perfusion of the SAN to meet its unique metabolic demands[61]. Studies have suggested a correlation between coronary artery diseases involving the SANa and supraventricular tachycardias[11,43]. One study found a 50% reduction in capillary density in the SAN among patients with atrial fibrillation compared to patients with sinus rhythm[62]. As such, tissue ischemia and fibrosis secondary to the SANa occlusion may lead to SAN dysfunction[63], although further studies are required to establish such correlation. Furthermore, the role of SANa variations in the development of atrial pathologies is yet to be explored.

**Fig 2:**
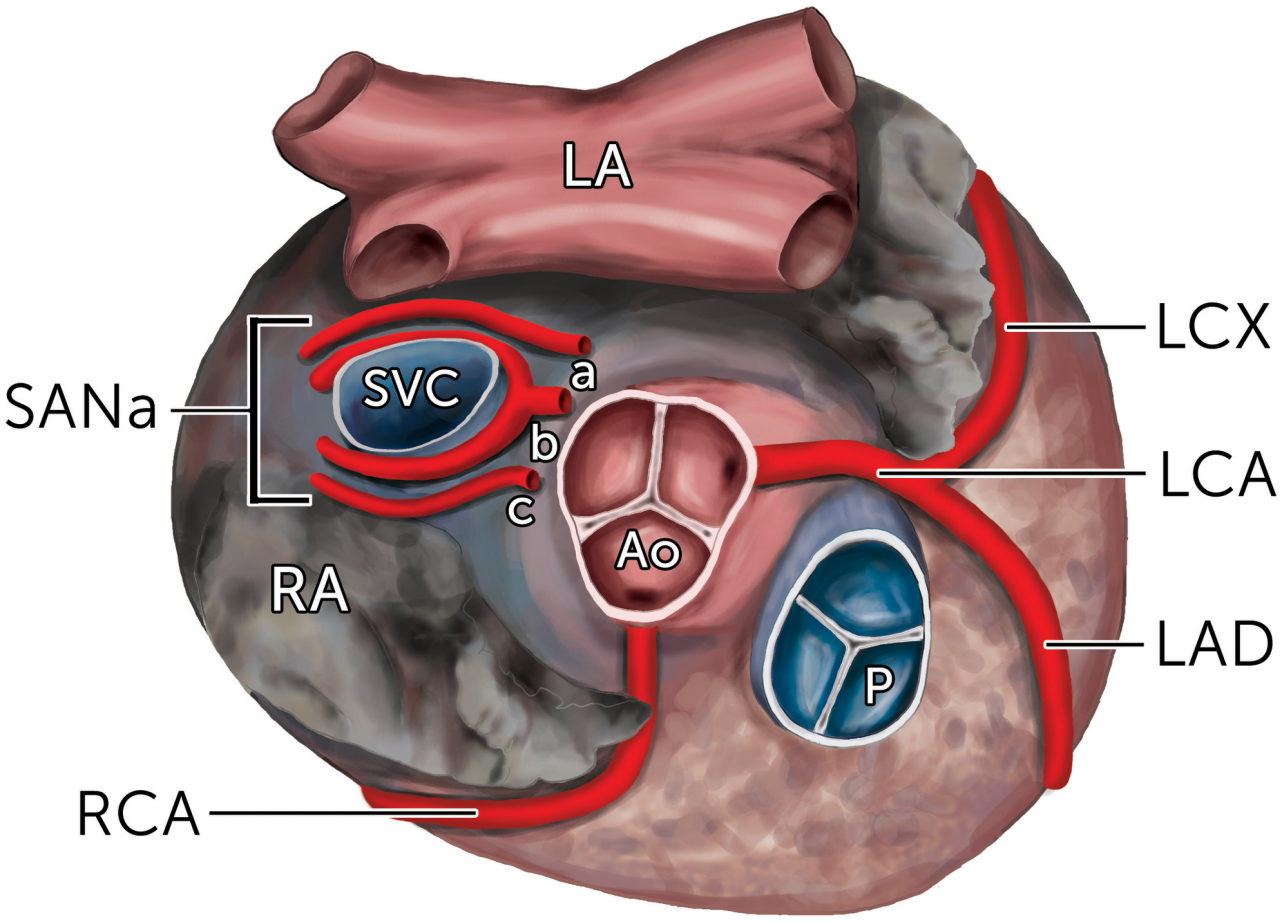
The various courses of the sinoatrial nodal artery (SANa) as seen from a superior view. (A) Retrocaval course; (B) Pericaval course; (C) Precaval course. LA, Left Atrium; RA, Right Atrium; SVC, Superior Vena Cava; Ao, Aorta; P, Pulmonary Trunk; RCA, Right Coronary Artery; LCA, Left Coronary Artery; LCX, Left Circumflex Artery; LAD, Left Anterior Descending Artery; SANa, Sinoatrial Nodal Artery.

Variations in the course of the SANa in relation to the superior vena cava have also been reported. The different courses of the SANa after branching is termed by its trajectory in relation to the superior vena cava (SVC). The artery can run in precaval (anterior to the SVC), retrocaval (posterior to the SVC), or pericaval (combination of pre- and retrocaval) courses (Fig 2). Furthermore, a rare S-shaped course of the SANa has also been described, which is reportedly susceptible to an increased risk of iatrogenic injury during cardiac procedures due to its irregular course and proximity to the left atrial wall[50].

As the use of percutaneous and surgical therapy for supraventricular arrhythmias is increasing[64], a thorough evidence-based knowledge of the anatomy of the SANa is essential for clinical practice[35,37,54]. As such, the aim of our study was to determine the normal anatomy of the SANa and the prevalence of its variations in the population using a meta-analytical approach.

## Materials and Methods

### Search Strategy

In order to identify articles for inclusion in the meta-analysis, we performed a literature search through June 2015 of the major electronic databases PubMed, EMBASE, Science Direct, Scopus, Web of Science, Cochrane Library, and China National Knowledge Infrastructure (CNKI). To achieve a high sensitivity among the search results, the search strategy was particularly tailored for each database. No lower date limit or language restrictions were applied. The following search term combination was used to search the electronic databases: “sinus node artery” OR “Sinus Node Branch” OR “Keith-flack node” OR “SA Node artery” OR “SAN Node branch” OR “SAN artery” OR “SAN branch” OR “sinus node blood supply” OR “sinoatrial artery” OR “sinoatrial branch” OR “sinoatrial blood supply” OR “sinuatrial artery” OR “sinuatrial branch” OR “sinuatrial blood supply” OR “Sinus Node vasculature” OR “SA Node vasculature” OR "SA Node blood supply."

To identify additional articles eligible for the analysis, the references of all articles included in the meta-analysis were extensively searched. Case reports, conference abstracts, and letters to the editor were searched but not included in the meta-analysis. The authors strictly followed the Preferred Reported Items for Systematic Reviews and Meta-analyses (PRISMA) guidelines were strictly followed throughout the search process and the entire meta-analysis (S1 Table) [65].

### Criteria for Study Selection

Each study was independently assessed by three reviewers (J.V., B.M.H., and W.C.H.) for eligibility in the meta-analysis. Studies were considered eligible for inclusion if they (1) reported data on the anatomy of the SANa in humans, (2) were a cadaveric or imaging study, and (3) had clearly defined anatomical definitions. The exclusion criteria for studies were (1) missing or incomplete data, (2) unclear anatomical definitions, or (3) the study included patients with congenital heart diseases or malformations. All relevant studies that were published in languages not fluently spoken by any of the authors were translated by medical professionals, fluent in both English and the language of the original article. When necessary, authors of the original study were contacted, if possible, for further details or data. Any disagreements among the authors during the eligibility assessment process were solved by a consensus among the entire review team.

### Data Extraction

Data were independently extracted from the included studies by two reviewers (J.V. and W.C.H.). The extracted data included study design, modality, country, sample size, number of SANa (single, duplication, or triplication), origin of the SANa, course of the SANa, the distance from the ostium to the SANa, diameter of the SANa, and the prevalence of S-shaped branches. In the event of any discrepancies in the data, the authors of the original study were contacted, if possible, for further information.

### Statistical Analysis

Statistical analysis was performed by B.M.H. and P.K.R. using MetaXL version 2.0 by EpiGear International Pty Ltd (Wilston, Queensland, Australia) to calculate the single or multicategorical pooled prevalence of an anatomical characteristic of the SANa[65]. Data on mean lengths and diameters of the SANa were pooled into a meta-analysis using Comprehensive Meta-Analysis version 3.0 by Biostat (Englewood, New Jersey, USA). A random effects model was used to perform all analyses. In order to assess the heterogeneity among the included studies, the Chi^2^ test and I^2^ statistic were calculated. For the Chi^2^ test, a p-value of <0.10 was considered to indicate significant heterogeneity between studies[66]. For the I^2^ statistic, the value was interpreted according to the following: 0% to 40% might not be important; 30% to 60% may represent moderate heterogeneity; 50% to 90% may represent substantial heterogeneity; and 75% to 100% may represent considerable heterogeneity[66].

When appropriate, to explore the sources of heterogeneity, subgroup analysis based on the type of study or geographical origin of the study was performed. Statistically significant differences between 2 or more groups/subgroups were determined through the use of confidence intervals. If the confidence intervals between two groups/subgroups overlapped, the differences were not considered statistically significant. Additionally, to further probe the source of heterogeneity, a sensitivity analysis was also performed when appropriate by limiting inclusion to studies with ≥ 100 hearts.

## Results

### Study Identification

**Fig 3:**
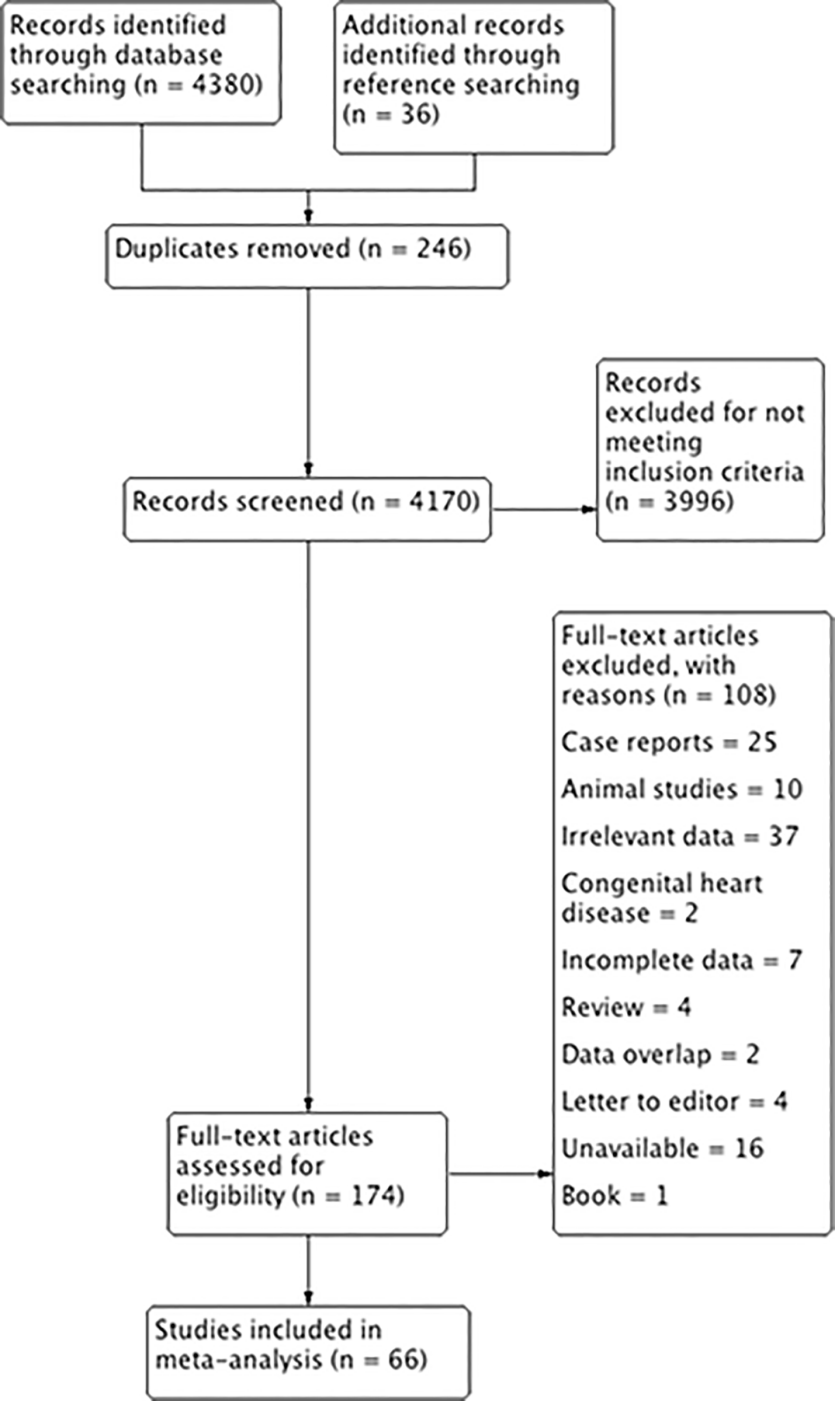
Flow chart of study identification, evaluation and inclusion in the meta-analysis.

An overview of the study identification process is summarized in the PRISMA flow diagram (Fig 3). A total of 4380 articles were initially identified by searching all major electronic databases, with an additional 36 articles identified via searching the references of included articles for any other potentially eligible articles. Subsequently, 174 articles were assessed by full-text for eligibility in the meta-analysis following screening and removal of duplicates. Of the 174 full-text articles, 108 articles were excluded due to specific reasons and 66 were deemed eligible for inclusion in the meta-analysis.

### Characteristics of Included Studies

The characteristics of included studies are summarized in Table 1. In the total of 66 studies (n = 21455 hearts) included in the meta-analysis, there were 42 cadaveric studies[3,5–8,10,11,13,14,16–22,27–31,35–38,40–42,44,45,48,51,52,54,56,59,67–72] and 24 imaging studies[9,12,15,23–26,32–34,39,43,46,47,49,50,53,55,57,58,73–76]. The modalities used in these imaging studies were either X-ray angiography (6 studies)[34,39,43,47,74,76] or Computed Tomography (CT) angiography (18 studies)[9,12,15,23–26,32,33,46,49,50,53,55,57,58,73,75]. The reported characteristics of the patient population varied between the included studies. In the cadaveric studies, the causes of death amongst the patients were mostly unspecified, but were also due to various accidents or coronary artery diseases (CAD) (5 studies). In the imaging studies, most of the patients had a suspected or known CAD or other cardiac pathologies (16 studies). A wide geographical distribution was observed in the included studies, with a majority of studies originating from China (14 studies), USA (8 studies), Turkey (6 studies), Greece (5 studies), France (4 studies), and Brazil (3 studies). The number of hearts assessed in the included studies also demonstrated a wide variation, ranging from 10 to 3802. Since no lower date limit was set in the meta-analysis, the dates of the included studies ranged from 1958 to 2014.

**Table 1:** Characteristics of the included studies.

Study	Type	Country	n =	From RCA (%)	From LCX (%)	From LCA (%)	Double Origin (%)	Other (%)
Bokeriia 2014[73]	I (MSCT-A)	Russia	38	84.21	15.79	0	0	0
Shimotakahara 2014[51]	C	Japan	293	56.31	36.18	0	7.51	0
Chi 2013[32]	I (CT-A)	China	196	59.18	0	0	5.61	35.20
Dong 2013[33]	I (320 CT)	China	3082	54.15	41.34	0	4.51	0
Cezlan 2012[25]	I (64 CT)	Turkey	400	58.25	37.25	0	4.25	0.25
Erol 2012[9]	I (64 CT)	Turkey	2072	65.59	33.69	0.05	0	0.68
Song 2012[53]	I (CT)	Korea	496	53.43	42.94	0	3.63	0
Tian 2012[55]	I (CT-A)	China	155	63.23	23.87	0	12.90	0
Yokokawa 2012[75]	I (CT)	USA	74	70.27	29.73	0	0	0
Ballesteros 2011[29]	C	Colombia	221	60.63	34.84	0	4.52	0
Nerantzis 2011[42]	C	Greece	200	59.00	39.00	0	2.00	0
Andreini 2010[23]	I (64 CT)	Italy	2495	73.71	16.91	0	8.50	0.88
Ballesteros 2010[28]	C	Colombia	88	59.10	37.50	0	3.41	0
Nerantzis 2010[41]	C	Greece	400	61.25	36.75	0	2.00	0
Ou 2010[15]	I (CT-A)	China	1704	52.93	0.53	46.54	0	0
Ozturk 2011[26]	I (64 CT)	Turkey	251	55.38	39.44	0	3.98	1.20
Yildirim 2010[57]	I (CT)	Turkey	107	96.26	3.74	0	0	0
Kosar 2009[12]	I (64 CT)	Turkey	700	79.14	20.00	0.43	0	0.43
Okmen 2009[43]	I (A)	Turkey	1500	86.13	13.87	0	0	0
Peng 2009[46]	I (CT-A)	China	272	57.35	37.50	0	5.15	0
Ramanathan 2009[47]	I (A)	India	300	53.00	42.67	0	4.33	0
Cademartiri 2008[24]	I (64 CT)	Italy	497	71.42	18.11	0	10.26	0.20
Pejkovic 2008[16]	C	Slovenia	150	63.33	35.33	1.33	0	0
Saremi 2008[50]	I (64 CT)	USA	101	66.34	27.72	0	5.94	0
Saremi 2008a[49]	I (64 CT)	USA	244	54.51	40.57	0	4.92	0
Zhang 2008[58]	I (CT)	China	96	45.83	43.75	0	10.42	0
Onciu 2006[44]	C	Romania	50	74.00	16.00	0	10.00	0
Ortale 2006[45]	C	Brazil	50	50.00	44.00	0	4.00	2.00
Li 2005[74]	I (A)	China	511					
Berdajs 2003[3]	C	Hungary	50	66.00	34.00	0	0	0
Futami 2003[35]	C	Japan	30	73.33	3.33	0	23.33	0
Kawashima 2003[37]	C	Japan	106	32.08	14.15	0	50.00	3.77
Song 2002[52]	C	China	80	65.00	33.75	0	1.25	0
Sow 1996[54]	C	Senegal	45	64.44	24.44	0	11.11	0
Caetano 1995[6]	C	Brazil	100	58.00	30.00	12.00	0	0
Didio 1995[8]	C	Brazil	100	58.00	0	42.00	0	0
Yangni-Angate 1995[21]	C	Ivory Coast	43	46.51	0	23.26	30.23	0
Yao 1995[71]	C	China	150					
Ndiaye 1994[14]	C	Senegal	15	40.00	0	13.33	0	46.67
Krupa 1993[38]	C	Poland	114	53.51	46.49	0	0	0
Eliska 1990[72]	C	Czech Republic	60					
Kyriakidis 1988[39]	I (A)	Greece	309	58.90	38.51	0	2.59	0
Sahni 1988[70]	C	India	100	73.00	0	0	0	27.00
Wei 1987[19]	C	China	1326	58.67	0	41.10	0.23	0
Bokeriya 1984[5]	C	Russia	70	61.43	28.57	10.00	0	0
Busquet 1984[30]	C	France	50	66.00	30.00	0	4.00	0
Xiao 1984[20]	C	China	41	80.49	0	7.32	12.20	0
Nerantzis 1983[76]	I (X Ray & Corrosion Casting)	Greece	360	61.94	38.10	0	0	0
Shi 1998[17]	C	China	50	48.00	50.00	2.00	0	0
Nerantzis 1980[40]	C	Greece	300	61.67	37.00	0	1.33	0
Yu 1980[22]	C	China	50	52.00	0	48.00	0	0
Anderson 1979[67]	C	England	25					
Chen 1978[31]	C	China	30	80.00	20.00	0	0	0
Hutchinson 1976[36]	C	UK	40	65.00	35.00	0	0	0
Christides 1975[7]	C	France	60	66.67	0	26.67	6.67	0
Engel 1975[34]	I (CA)	USA	80	60.00	38.75	0	1.25	0
Vieweg 1975[18]	C	USA	118	53.39	0	34.75	11.86	0
Kennel 1972[69]	C	USA	70	65.71	34.29	0	0	0
Lienhard 1972[13]	C	France	25	68.00	0	24.00	8.00	0
Hromada 1970[10]	C	Czech Republic	100	65.00	0	33.00	1.00	1.00
Hromada 1969[27]	C	Czech Republic	80	62.50	35.00	0	1.25	1.25
Mandard 1968[59]	C	France	100	64.00	36.00	0	0	0
Romhilt 1968[48]	C	USA	186	60.75	37.63	0	1.61	0
Verhaeghe 1967[56]	C	Belgium	10	40.00	50.00	0	10.00	0
James 1958[11]	C	USA	39	61.54	0	38.46	0	0
Crainicianu 1922[68]	C	Romania	200	68.00	7.00	0	25.00	0

SANa, Sinoatrial nodal artery; RCA, Right coronary artery; LCX, Left circumflex artery; LCA. Left coronary artery; C, Cadaveric; I, Imaging, CT-A, CT-Angiography; A, Angiography; MSCT-A, Multislice CT-Angiography; CA, Coronary Arteriography; PM-A, Post-mortem Angiography

### Number of the Sinoatrial Nodal Artery

A total of 53 studies (n = 17675 hearts) reported data on the number of the SANa[3,7,8,10–13,15–59,72]. The results are summarized in Table 2. The SANa was most commonly found to be present as a single vessel with a pooled prevalence of 95.5% (95%CI:93.6–96.9). Rarely, duplication and triplication of the artery were observed with a pooled prevalence of 4.3% (95%CI:2.8–6.0) and 0.3% (95%CI: 0–0.7), respectively. Subgroup analyses based on geographical distribution and type of study (cadaveric or imaging), as well as a sensitivity analysis (inclusive of all studies with ≥ 100 hearts), were performed to explore the high level of heterogeneity (l^2^ = 95.77%). The results of subgroup and sensitivity analyses are also summarized in Table 2 (S1 Fig).

**Table 2:** Number of the sinoatrial nodal artery (SANa) in different population subgroups.

Population	Number of studies (Number of hearts)	Single SANa (95% CI)	Double SANa (95% CI)	Triple SANa (95% CI)	I^2^ (95% CI)	Cochran's Q, P-value
All	53 (17675)	95.5 (93.6–96.9)	4.3 (2.8–6.0)	0.3 (0–0.7)	95.77 (95.04–96.38)	0.000
Africa	2 (88)	80.6 (59.1–96.5)	18.8 (3.5–40.9)	0.6 (0–6.9)	79.51 (11.55–95.26)	0.027
Asia	17 (8312)	94.8 (90.0–97.0)	4.9 (2.2–8.6)	0.03 (0–1.4)	97.12 (96.31–97.76)	0.000
Europe	24 (8048	96.6 (94.2–98.7)	3.2 (1.3–5.8)	0.2 (0–1.0)	96.15 (95.16–96.94)	0.000
North America	6 (768)	95.9 (92.6–98.5)	3.9 (1.5–7.4)	0.2 (0–1.2)	74.77 (42.76–88.88)	0.001
South America	4 (459)	97.0 (93.3–99.5)	2.6 (0.4–6.2)	0.5 (0–2.1)	69.13 (10.87–89.31)	0.021
Cadaveric	34 (4690)	94.9 (91.5–97.2)	4.6 (2.3–7.7)	0.5 (0–1.5)	93.99 (92.51–95.18)	0.000
Imaging	19 (12985)	96.3 (94.0–98.3)	3.6 (1.7–6.0)	0.1 (0–0.6)	97.35 (96.66–97.89)	0.000
Sensitivity Analysis (n ≥ 100)	32 (16523)	96.5 (94.7–98.1)	3.3 (1.9–5.2)	0.1 (0–0.6)	96.99 (96.38–97.50)	0.000

SANa, Sinoatrial nodal artery

### Origin of the Sinoatrial Nodal Artery

A total of 61 studies (n = 20721 hearts) reported data on the origin of the SANa[5–59,68–70,73,75,76]. The results are summarized in Table 3 (S2 Fig). The SANa was most commonly found to originate from the RCA with a pooled prevalence of 68.0% (95%CI:55.6–68.9). The second most common origin of the artery was from the LCX with a pooled prevalence of 22.1% (95%CI:15.0–26.2), followed by origin of the artery from the LCA with a pooled prevalence of 2.7% (95%CI:0.7–5.2). Other types of origin of the SANa such as from the aorta or the bronchial artery were equally rare with a pooled prevalence of 0.3% (95%CI:0–1.3). Although the prevalence of a double origin of the SANa was rare, when present, the artery most commonly originated from the RCA and LCX with a pooled prevalence of 2.0% (95%CI:0.3–4.2). The second most common type of double origin of the SANa was from the RCA and LCA with a pooled prevalence of 0.9% (95%CI:0–2.3). Other types of double origin of the SANa (i.e. from the RCA and bronchial artery, from the LCX and pulmonary artery, from the LCX and bronchial artery, both from the RCA, both from the LCX, or both from the LCA) were equally rare with a pooled prevalence of 0.3% (95%CI:0–1.3). The origin of the SANa when it was triplicated (i.e. 2 from the LCX + 1 from RCA, 2 from RCA + 1 from LCA, 2 from RCA + 1 from bronchial artery, or 1 from RCA + 2 from bronchial artery), or when it arose from the coronary sinus (left or right) were also similarly rare with a pooled prevalence of 0.3% (95%CI:0–1.2).

**Table 3:** Origin of the sinoatrial nodal artery (SANa) in different population subgroups.

Population	All	Africa	Asia	Europe	North America	South America	Cadaveric	Imaging	Sensitivity Analysis (n ≥ 100)
Number of Studies(Number of hearts)	61 (20721)	3 (103)	18 (8469)	27 (10678)	8 (912)	5 (559)	38 (5130)	23 (15591)	36 (19417)
From RCA(95% CI)	68.0(55.6–68.9)	54.8(18.7–83.0)	69.2(40.7–76.2)	69.4(61.8–72.6)	65.7(44.0–78.2)	61.4(30.3–82.9)	66.5(50.5–70.4)	68.5(55.3–74.4)	70.2(53.4–71.0)
From LCX(95% CI)	22.1(15.0–26.2)	6.4(0–26.2)	17.9(4.2–30.7)	24.6(19.1–28.9)	22.9(8.6–37.8)	25.4(4.4–50.5)	18.6(9.9–25.3)	26.9(17.3–34.8)	23.7(14.1–29.0)
From LCA(95% CI)	2.7(0.7–5.2)	11.0(0–34.3)	3.8(0–11.1)	1.1(0.1–2.6)	4.0(0–11.9)	6.3(0–21.7)	5.1(1.1–10.0)	0.5(0–2.3)	1.6(0–4.6)
From Aorta(95% CI)	0.3(0–1.3)	0.9(0–12.3)	0.2(0–3.6)	0.3(0–1.1)	0.3(0–3.7)	0.3(0–6.8)	0.4(0–2.1)	0.2(0–1.6)	0.2(0–1.3)
From Bronchial artery(95% CI)	0.3(0–1.2)	0.9(0–12.3)	0.2(0–3.6)	0.3(0–1.0)	0.3(0–3.7)	0.3(0–6.8)	0.4(0–2.2)	0.1(0–1.3)	0.1(0–1.2)
From RCA and LCX(95% CI)	2.0(0.3–4.2)	0.9(0–12.3)	4.4(0–12.1)	1.0(0.1–2.5)	2.9(0–9.9)	2.3(0–13.5)	2.0(0–5.0)	2.1(0–6.1)	2.2(0.1–5.5)
From RCA and LCA(95% CI)	0.9(0–2.3)	13.7(0–38.4)	0.6(0–4.8)	0.7(0–2.0)	0.3(0–3.7)	0.3(0–6.8)	1.6(0–4.5)	0.2(0–1.5)	0.3(0–1.7)
From RCA and Bronchial artery(95% CI)	0.3(0–1.2)	0.9(0–12.3)	0.2(0–3.6)	0.2(0–0.9)	0.3(0–3.7)	0.3(0–6.8)	0.4(0–2.1)	0.1(0–1.3)	0.1(0–1.1)
From LCX and Pulmonary artery(95% CI)	0.3(0–1.2)	0.9(0–12.3)	0.3(0–3.7)	0.2(0–0.9)	0.3(0–3.7)	0.3(0–6.8)	0.4(0–2.2)	0.1(0–1.4)	0.1(0–1.2)
From LCX and Bronchial artery(95% CI)	0.3(0–1.2)	0.9(0–12.3)	0.2(0–3.6)	0.2(0–0.9)	0.3(0–3.7)	0.3(0–6.8)	0.4(0–2.1)	0.1(0–1.3)	0.1(0–1.1)
2 from RCA(95% CI)	0.3(0–1.3)	0.9(0–12.3)	0.4(0–4.2)	0.2(0–1.0)	0.3(0–3.7)	0.3(0–6.8)	0.5(0–2.5)	0.1(0–1.3)	0.2(0–1.3)
2 from LCX(95% CI)	0.3(0–1.2)	0.9(0–12.3)	0.4(0–4.0)	0.2(0–0.8)	0.3(0–3.7)	0.3(0–6.8)	0.5(0–2.3)	0.1(0–1.3)	0.2(0–1.2)
2 from LCA(95% CI)	0.3(0–1.1)	0.9(0–12.3)	0.2(0–3.6)	0.2(0–0.8)	0.3(0–3.7)	0.3(0–6.8)	0.4(0–2.1)	0.1(0–1.3)	0.1(0–1.1)
2 from RCA + 1 from LCX(95% CI)	0.3(0–1.2)	0.9(0–12.3)	0.3(0–3.7)	0.2(0–0.8)	0.3(0–3.7)	0.3(0–6.8)	0.4(0–2.2)	0.1(0–1.3)	0.1(0–1.2)
2 from LCX + 1 from RCA(95% CI)	0.3(0–1.2)	0.9(0–12.3)	0.3(0–3.9)	0.2(0–0.8)	0.3(0–3.7)	0.3(0–6.8)	0.4(0–2.2)	0.1(0–1.3)	0.1(0–1.2)
2 from RCA + 1 from LCA(95% CI)	0.3(0–1.1)	0.9(0–12.3)	0.2(0–3.6)	0.2(0–0.8)	0.3(0–3.7)	0.3(0–6.8)	0.4(0–2.1)	0.1(0–1.3)	0.1(0–1.1)
2 from RCA + 1 from Bronchial artery(95% CI)	0.3(0–1.2)	0.9(0–12.3)	0.2(0–3.6)	0.2(0–0.8)	0.3(0–3.7)	0.3(0–6.8)	0.4(0–2.2)	0.1(0–1.3)	0.1(0–1.1)
1 from RCA + 2 from Bronchial artery(95% CI)	0.3(0–1.1)	0.9(0–12.3)	0.2(0–3.6)	0.2(0–0.8)	0.3(0–3.7)	0.5(0–8.0)	0.4(0–2.1)	0.1(0–1.3)	0.1(0–1.1)
From Right Coronary sinus(95% CI)	0.3(0–1.2)	0.9(0–12.3)	0.2(0–3.6)	0.2(0–0.9)	0.3(0–3.7)	0.3(0–6.8)	0.4(0–2.1)	0.1(0–1.3)	0.1(0–1.1)
From Left Coronary sinus(95% CI)	0.3(0–1.2)	0.9(0–12.3)	0.2(0–3.6)	0.2(0–0.9)	0.3(0–3.7)	0.3(0–6.8)	0.4(0–2.1)	0.1(0–1.3)	0.1(0–1.1)
I^2^(95% CI)	98.94(98.84–99.03)	89.75(72.46–96.19)	99.57(99.51–99.62)	96.71(95.96–97.32)	96.33(94.49–97.56)	0.3(0–6.8)	98.02(97.71–98.29)	99.32(99.23–99.41)	99.35(99.28–99.41)
Cochran's Q, P-value	0.000	0.000	0.000	0.000	0.000	0.000	0.000	0.000	0.000

SANa, Sinoatrial nodal artery; RCA, Right coronary artery; LCX, Left circumflex artery; LCA, Left coronary artery

Due to the significant heterogeneity found in the analysis (l^2^ = 98.94%), subgroup analyses were performed dependent on the geographical distribution and type of study. The results of the subgroup analyses and an additional sensitivity analysis are summarized in Table 3.

### Course of the Sinoatrial Nodal Artery

A total of 19 studies (n = 6033 hearts) reported data on the course of the SANa[3,10,17,22,25–28,30,33,46,49–51,53,54,58,67,76]. With a pooled prevalence of 47.1% (95%CI:36.0–55.5), the retrocaval course of the SANa was the most common course of the artery, followed by the precaval and pericaval courses with a pooled prevalence of 38.9% (95%CI:28.5–47.6) and 14.0% (95%CI:7.5–21.1), respectively. Subgroup analyses based on the geographical distribution and type of study, and a sensitivity analysis were performed because of the high level of heterogeneity found (l^2^ = 97.66%). The results are summarized in Table 4 (S3 Fig).

**Table 4:** Course of the sinoatrial nodal artery (SANa) in different population subgroups.

Population	Number of Studies (Number of hearts)	Precaval (95% CI)	Retrocaval (95% CI)	Pericaval (95% CI)	I^2^(95% CI)	Cochran's Q, P-value
*Course of the SANa in Total*						
All	19(6033)	38.9(28.5–47.6)	47.1(36.0–55.5)	14.0(7.5–21.1)	97.66 (97.08–98.13)	0.000
Asia	7(4453)	28.6(14.4–42.8)	55.2(37.0–68.6)	16.1(5.5–28.8)	98.39 (97.74–98.85)	0.000
Europe	8(1316)	41.8(25.9–55.7)	47.3(30.8–60.9)	10.9(2.7–21.9)	96.27(94.38–97.52)	0.000
North America	2(135)	52.4(30.7–74.0)	38.9(18.8–61.1)	8.7(0–23.0)	79.71(12.52–95.29)	0.026
Cadaveric	10(852)	43.4(25.4–58.9)	41.3(23.6–56.8)	15.3(4.4–29.0)	95.52(93.43–96.94)	0.000
Imaging	9(5181)	34.4(21.4–46.6)	53.6(38.7–65.3)	12.0(4.2–21.7)	98.40(97.86–98.81)	0.000
Sensitivity Analysis(n ≥ 100)	10(5567)	35.8(21.3–48.3)	51.9(35.5–63.8)	12.3(3.9–22.6)	98.79(98.44–99.06)	0.000
*Course of the SANa from the left (i*.*e*. *LCA or LCX)*						
All	9(651)	37.3(22.2–53.4)	46.5(30.4–62.5)	16.2(5.8–29.9)	93.17(89.18–95.69)	0.000
Asia	3(418)	40.2 (4.6–77.4)	44.9 (7.0–81.1)	14.9 (0–45.6)	98.0 (96.29–98.92)	0.000
Europe	4(186)	28.5(5.6–61.6)	56.3(28.0–87.5)	15.2(0–41.5)	92.60(84.28–96.52)	0.000
Cadaveric	6(261)	40.6(17.9–64.7)	47.5(23.6–71.2)	12.0(0.3–32.3)	91.60(84.48–95.45)	0.000
Imaging	3(390)	30.4(21.7–39.5)	45.8(36.1–55.4)	23.8(15.9–32.5)	67.98(0–90.72)	0.044
Sensitivity Analysis(n ≥ 100)	3(475)	38.4(7.4–70.9)	49.3(14.5–80.0)	12.2(0–37.0)	97.92(96.12–98.89)	0.000
*Course of the SANa from the RCA*						
All	9(893)	43.1(25.3–58.3)	38.7(21.5–54.0)	18.2(6.3–32.2)	95.67(93.53–97.10)	0.000
Asia	3(518)	33.7(0–74.9)	52.2(7.5–91.3)	14.1(0–49.7)	98.70(97.77–99.24)	0.000
Europe	4(293)	45.1(28.9–60.1)	34.2(19.5–49.2)	20.7(9.0–34.5)	84.47(61.15–93.80)	0.000
Cadaveric	6(413)	55.0(26.0–77.3)	29.9(7.6–54.3)	15.0(0.5–37.5)	95.84(93.16–97.47)	0.000
Imaging	3(480)	23.3(10.2–38.4)	54.1(36.2–69.5)	22.6(9.7–37.7)	90.95(76.41–96.53)	0.000
Sensitivity Analysis(n ≥ 100)	3(611)	41.3(6.0–76.7)	45.7(8.5–80.2)	13.0(0–41.3)	98.69(97.75–99.24)	0.000

SANa, Sinoatrial nodal artery; RCA, Right coronary artery; LCX, Left circumflex artery; LCA, Left coronary artery

Nine of the studies above also reported data on the course of the SANa originating from the left (i.e. from the LCA or LCX) or the right (i.e. the RCA) side of the heart. In the nine studies (n = 651 hearts) reporting data on the course of the SANa originating from the LCA or LCX[3,10,26–28,51,53,54,58], the retrocaval course of the SANa was also the most common course of the artery with a pooled prevalence of 46.5% (95%CI:30.4–62.5). On the contrary, analysis on the nine studies (n = 893 hearts) reporting the course of the SANa originating from the RCA[3,10,26–28,51,53,54,58] found the precaval course to be the most common course of the SANa with a pooled prevalence of 43.1% (95%CI:25.3–58.3). Subgroup analyses in regards to the geographical distribution and type of study, as well as a sensitivity analysis were also performed. The results for the course of the SANa originating from the LCA or LCX and RCA are summarized in Table 4.

### S-Shaped Branches of the Sinoatrial Nodal Artery

Eight studies (n = 5031 hearts) reported data on the prevalence of S-shaped branches of the SANa, The pooled prevalence of the S-shaped branched of the artery was merely 7.6% (95%CI:2.9–14.1). Subgroup analyses by the geographical distribution and type of study were also performed, results of which are summarized in Table 5 (S4 Fig).

**Table 5:** S-shaped branches of the sinoatrial nodal artery (SANa) in different population subgroups.

Population	Number of Studies (Number of hearts)	S-shaped branches (95% CI)	I^2^(95% CI)	Cochran's Q, P-value
All	8(5031)	7.6(2.9–14.1)	97.24 (95.99–98.09)	0.000
Asia	2 (3640)	1.3 (0.5–2.5)	71.94 (0–93.69)	0.059
Europe	3 (951)	10.4 (3.9–19.3)	93.15 (83.33–97.18)	0.000
North America	2 (352)	9.5 (3.1–18.7)	80.21 (15.05–95.39)	0.025
Imaging	6 (4643)	6.4 (1.5–13.8)	97.64 (96.42–98.45)	0.000

### Morphometric Measurements of the Sinoatrial Nodal Artery

A total of 18 studies (n = 3313 RCA) measured the distance between the origin of SANa and the ostium of RCA[16,17,25,26,28,29,32,33,45,46,49,52,54–56,58,71,74], while a total of 14 studies (n = 2029 LCX) measured the distance between the origin of SANa and the ostium of LCX[17,25,26,28,32,33,45,46,49,50,52,55,56,58]. The pooled mean distance from the ostium of RCA to the origin of SANa and the ostium of LCX to the origin of SANa was 16.306 mm (95%CI:14.134–18.478) and 14.323 mm (95%CI:11.706–16.939), respectively. Subgroup analyses based on the geographical distribution and type of study were also performed. The results are summarized in Table 6.

**Table 6:** Distance between the origin of the sinoatrial nodal artery (SANa) and the ostia of the right coronary artery (RCA) and the left circumflex artery (LCX) in different population subgroups.

Population	Number of studies (Number of RCA / LCX)	Pooled mean, mm (95%CI)	I^2^	Cochran’s Q, P-value
*Distance from the ostium of the RCA to the origin of the SANa*				
All	18 (3313)	16.306 (14.134–18.478)	98.01	0.000
Asia	9 (2516)	16.308 (12.796–19.819)	98.99	0.000
Europe	4 (480)	15.977 (12.178–19.775)	94.28	0.000
South America	3 (221)	16.025(14.626–17.424)	0.00	0.542
Cadaveric	9 (499)	15.041 (13.270–16.812)	82.46	0.000
Imaging	9 (2814)	17.305 (13.918–20.693)	98.97	0.000
*Distance from the ostium of the LCX to the origin of the SANa*				
All	14 (2029)	14.323 (11.706–16.939)	98.40	0.000
Asia	7 (1585)	10.359 (7.746–12.972)	98.37	0.000
Europe	3 (262)	20.360 (12.948–27.772)	92.45	0.000
North America	2 (127)	20.337 (3.971–36.702)	98.92	0.000
South America	2 (55)	14.164 (10.832–17.496)	0.00	0.578
Cadaveric	5 (112)	10.867 (6.783–14.951)	83.24	0.000
Imaging	9 (1917)	15.893 (12.582–19.203)	98.59	0.000

SANa, Sinoatrial nodal artery; RCA, Right coronary artery; LCX, Left circumflex artery

The diameter of the SANa (originating from the RCA) at its origin and within the SAN were reported by seven (n = 587 hearts)[16,25,28,29,45,56,58] and six (n = 2315 hearts)[32,33,45,46,71,74] studies, respectively. At its origin, the pooled mean diameter of the SANa originating from the RCA was 1.349 mm (95%CI:1.185–1.512). Within the SAN, the pooled mean diameter of the SANa originating from the RCA was 1.457 mm (95%CI:1.217–1.697).

Seven (n = 381 hearts)[16,25,28,29,45,56,58] and five (n = 1753 hearts)[32,33,45,46,74] studies also reported the diameter of the SANa (originating from the LCX) at its origin and within the SAN, respectively. At its origin, the pooled mean diameter of the SANa originating from the LCX was 1.481 mm (95%CI:1.124–1.837). Within the SAN, the pooled mean diameter of the SANa originating from the LCX was 1.400 mm (95%CI:1.049–1.750). Subgroup analyses based on the geographical distribution and type of study were also performed. The results are summarized in Table 7.

**Table 7:** Diameter of the sinoatrial nodal artery (SANa) in different population subgroups.

Population	Number of studies (Number of hearts)	Pooled mean, mm (95% CI)	I^2^	Cochran's Q, P-value
*Diameter of the SANa originating from the RCA at its origin*				
All	7 (587)	1.349 (1.185–1.512)	95.86	0.000
Europe	3 (332)	1.324 (0.884–1.764)	97.77	0.000
South America	3 (211)	1.384 (1.144–1.625)	96.35	0.000
Cadaveric	5 (310)	1.412 (1.191–1.633)	96.49	0.000
Imaging	2 (277)	1.194 (0.988–1.400)	91.74	0.001
*Diameter of the SANa originating from the RCA within the SAN*				
All	6 (2315)	1.457 (1.217–1.697)	99.52	0.000
Asia	5 (2290)	1.508 (1.247–1.769)	99.57	0.000
Cadaveric	2 (99)	1.349 (1.055–1.643)	95.82	0.000
Imaging	4 (2216)	1.510 (1.209–1.811)	99.68	0.000
*Diameter of the SANa originating from the LCX at its origin*				
All	7 (381)	1.481 (1.124–1.837)	99.13	0.000
Europe	3 (207)	1.484 (0.629–2.339)	99.69	0.000
South America	3 (132)	1.532 (1.306–1.757)	93.94	0.000
Cadaveric	5 (190)	1.608 (1.214–2.002)	98.89	0.000
Imaging	2 (191)	1.160 (0.895–1.424)	93.37	0.000
*Diameter of the SANa originating from the LCX within the SAN*				
All	5 (1753)	1.400 (1.049–1.750)	99.72	0.000
Asia	4 (1731)	1.474 (1.074–1.874)	99.77	0.000
Imaging	4 (1731)	1.474 (1.074–1.874)	99.77	0.000

SANa, Sinoatrial nodal artery; SAN, Sinoatrial node; RCA, Right coronary artery; LCX, Left circumflex artery

## Discussion

A normal heart rhythm refers to a regular rhythm with signal initiation from the SAN at an adequate heart rate. The crescent-shaped SAN is a collection of specialized myocytes located in the posterior wall of the right atrium, near the junction of the crista terminalis with the superior vena cava[67]. These myocytes are characterized by automaticity, which facilitates spontaneous depolarization of their cell membrane. Several studies have investigated the anatomical features of the SANa, which is the major blood supply to the SAN. The aim of our study was to pool data from all relevant studies to provide comprehensive, evidence-based anatomical data on the SANa with clinical implications for interventional cardiologists and cardiosurgeons.

Although rare, duplication and triplication of the SANa can occur in the human population[7,10,13,18,19,21,23–30,32–35,37,40–42,44–56,58,72] and should be taken into account by surgeons. The presence of such anatomical variations should be carefully evaluated before open heart surgery to prevent iatrogenic injury to one of the branches. Our analysis showed that SANa is represented by a single branch in 95.5% of cases, and the pooled prevalence of duplication and triplication were 4.3% and 0.3%, respectively. Subgroup analysis according to geography showed that a duplicated SANa has a higher prevalence in the African population (18.8%) compared to the European (3.2%), Asian (4.9%), North American (3.9%) and South American (2.6%) populations. There has been speculation in the literature regarding ethnical variations in the SANa anatomy[45]. However, due to the small sample sizes of the African studies in this meta-analysis (n = 88), larger studies will be needed to establish such geographical variation. Further subgroup analysis according to the type of study revealed that cadaveric studies reported a slightly higher pooled prevalence of duplicated (4.6%) and triplicated (0.5%) SANa when compared to imaging studies (3.6% and 0.1%, respectively), although the difference was not statistically significant.

The SANa was found to originate most commonly from the RCA (68.0%) with the LCX being the second most common point of origin (22.1%). Sensitivity analysis on the SANa origin yielded similar results for origin from the RCA and from the LCX with 70.2% and 23.7%, respectively. This is in agreement with previous reports in the literature[5,7,9,12,13,23,24,27–31,36,44,49,54,55,70,73] which revealed a predominance of left-sided origin of SANa. Among the included studies, only two(17,56) reported predominance of left-sided origin of SANa. However, both these studies had a small sample size. A single extra-coronary origin of SANa was found in 0.6% of cases, represented by aortic (0.3%) and bronchial artery (0.3%) origins. Sinoatrial nodal artery originating from the bronchial artery presents an interesting hemodynamic situation where the SANa is filled during systole, which in theory, would put these patients at risk of SAN ischemia, especially in cases with concomitant cardiac hypertrophy. In cases of a duplicated SANa, the most common anatomical pattern was one branch arising from the RCA and the other from the LCX (2.0%). It is reasonable to believe that such bi-coronary blood supply of SAN would prevent ischemia in case of vasoocclusive disease of one of the coronary arteries.

Only one of the included studies, by Ramanathan et al.[47], reported complete and accurate data on the origin of the SANa with respect to coronary artery dominance. They found that in a right coronary artery dominant heart, the SANa originated from the RCA in 49.4%, and from the LCX in 45.7%. However, in left coronary artery dominance, 63.2% of SANa originated from RCA, while only 36.8% originated from the LCX. In co-dominance, they found 51.5% originated from RCA and 41.2% from LCX. It may be suspected that coronary dominance affects the origin of the SANa, and as such, future studies should report SANa origin with respect to coronary dominance to further study the association between these two variables.

Knowledge about the course of SANa has clinical implications as the precaval course is considered to carry a lower risk of iatrogenic SANa injury during surgical incisions using the superior septal approach[2,3]. Overall, the most common course of SANa was found to be retrocaval (47.1%), followed by precaval (38.9%) and pericaval (14.0%). Similar pooled prevalences were found during subgroup analysis on SANa originating from the LCA and the LCX. Interestingly, when the SANa originated from the RCA, the precaval course was found to be the most common (43.1%) followed by the retrocaval (38.7%) and pericaval (18.2%) courses. This finding could be explained by the anatomical location of the RCA, as it usually branches off the ventral aspect of the aorta. Therefore, in order for the SANa originating from the RCA to take a retrocaval course to the SAN, it would require a developmental turn of nearly 180° to course posteriorly between the aorta and the SVC (Fig 1A). However, further subgroup analysis revealed that this precaval tendency was primarily noted in the cadaveric studies, whereas the imaging studies reported that a SANa from the RCA most commonly followed the retrocaval course (54.1%). This difference however, was not statistically significant. A retrocaval course was most prevalent in all populations, except North Americans, which showed a predominance of the precaval course (52.4%). The pericaval course was more often found in Asians (16.1%) than in Europeans (10.9%) and North Americans (8.7%), and was more often described in imaging than cadaveric studies, both for SANa from the RCA (22.6% vs. 15.0%) and from the LCX (23.8% vs. 12.0%).

Our data shows that S-shaped branches of SANa have a pooled prevalence of only 7.6%, which is lower than what has been reported in previous studies[39,50]. Geographical subgroup analysis showed a pooled prevalence of 10.4% in the European population and 9.5% in the North American population, while their prevalence in the Asian population was significantly lower (1.3%). Such S-shaped branches are of clinical significance as their long course makes them susceptible to iatrogenic damage during Cox maze operation for atrial fibrillation[50,77]. As such, cardiac surgeons must be aware of their existence and take special care in their presence to avoid iatrogenic injury.

The mean diameter of SANa arising from RCA was 1.349 mm at its origin, and 1.457 mm within SAN, the latter being larger than what has been reported previously[45]. While the SANa diameter was found in our analysis to be smaller at its origin than within the SAN itself, we suspect this to only be a statistical effect, due to the fact that a vast majority of studies only reported diameter at either the origin or within the SAN. In the one study which reported both diameters of an RCA arising SANa by Ortale et al.[45], the mean diameter at the origin (1.7 mm) was larger than that within the node (1.2 mm). In cases of SANa arising from the LCX, the pooled mean diameter was larger (1.481 mm), but this was not statistically significant. As artery diameter data may be useful for the planning of catheterization procedures, future studies should assess the diameter of the artery at multiple points from the origin to within the node.

This meta-analysis provides a solid, evidence-based description of the anatomical characteristics and variations of the SANa, which are essential to avoid iatrogenic injury during interventional cardiology (including ablation) and cardiosurgical (particularly open heart surgeries through the right atrium) procedures[26]. A detailed pre-operative knowledge of the SANa anatomy is crucial for optimizing cardio-invasive treatments[78]. Iatrogenic SANa injury, such as intra-operative damage (cross-clamping or dissection) of the artery as it crosses the superior posterior border of the interatrial septum during mitral valve surgery via the superior septal approach[79], may lead to additional postoperative arrhythmias[2,3]. Moreover, cardiac surgeons should be aware of the infeasibility of compensation for damage to the SANa that runs as a single vessel during operative procedures[43].

In percutaneous interventions such as angioplasty and stent implantation, especially in the proximal segment of RCA, distal embolization from the coronary artery to the proximally located SANa can cause severe bradycardia[43]. Additionally, as the SANa plays a major role in supplying blood to the atrial myocardium and atrial septum in up to 59% of individuals, any interruption of blood flow through the artery risks ischemia and infarction of these structures[41]. Therefore, understanding the normal and variant anatomy of the SANa can help interventionalists to prevent abrupt and adverse outcomes during invasive procedures. Lastly, a thorough knowledge of the arterial anatomy would also help interventional cardiologists in accurate interpretation of coronary angiograms[42].

Our meta-analysis was limited by the poor quality of some of the included studies, the high heterogeneity between studies, and the lack of available proper quality assessments for anatomical meta-analysis. Furthermore, due to the lack of an available proper measure for multi-categorical prevalence, no publication bias assessment was performed. In an attempt to minimize bias in our analysis, we contacted the original authors whenever necessary and possible in order to address any discrepancies in the data of the included studies.

Despite subgroup analysis by type of study and geographical distribution, as well as a sensitivity analysis, high heterogeneity persisted throughout the meta-analysis without any clear identifiable source. Through out the meta-analysis, no significant differences were found between studies on deceased patients (cadaveric studies) and living patients (imaging studies). We suspect that the primary source of the heterogeneity is the highly variable anatomical nature of the SANa itself. As such, we highly recommend physicians take care to identify the location and anatomical characteristics of the SANa prior to or during procedures, to reduce the risk of iatrogenic damage to the vessel.

With the anatomical basis from this meta-analysis, further studies should investigate the role of anatomical variations of SANa in different pathologies, including supraventricular tachycardias and sick sinus syndrome. Furthermore, studies should also investigate if certain SANa variations make patients with hypoxemia (i.e. COPD, pneumonia) more susceptible to development of atrial fibrillation.

## Conclusions

Although the anatomical characteristics of the SANa are highly variable, our comprehensive evidence-based assessment found that the most common form of the artery can be described as a single vessel, originating from the RCA, and taking a retrocaval course to reach the SAN. However, due to its high variability, physicians should be cautious in identifying the anatomy of the SANa in individual patients before or during interventional cardiology or cardiosurgical procedures. Furthermore, special attention should be paid to the relatively common S-shaped branch of the SANa, which exposes it to a particularly high risk of iatrogenic injury during such invasive procedures.

## Supporting Information

S1 Dataset Complete study data. (XLSX)

S1 Fig Forest plots showing pooled prevalence of the different number of SANa. (PDF)

S2 Fig Forest plots showing pooled prevalence of the different origins of SANa. (PDF)

S3 Fig Forest plots showing pooled prevalence of the different courses of SANa. (PDF)

S4 Fig Forest plot showing pooled prevalence of S-shaped SANa. (PDF)

S1 Table PRISMA 2009 Checklist. (DOCX)
