# Correction: Anatomical Variations in the Sinoatrial Nodal Artery: A Meta-Analysis and Clinical Considerations

**DOI:** 10.1371/journal.pone.0150051

**Published:** 2016-03-01

**Authors:** Jens Vikse, Brandon Michael Henry, Joyeeta Roy, Piravin Kumar Ramakrishnan, Wan Chin Hsieh, Jerzy A. Walocha, Krzysztof A. Tomaszewski

The image for Fig 1 is incorrect. Please see the corrected Fig 1 here.

**Fig 1:**
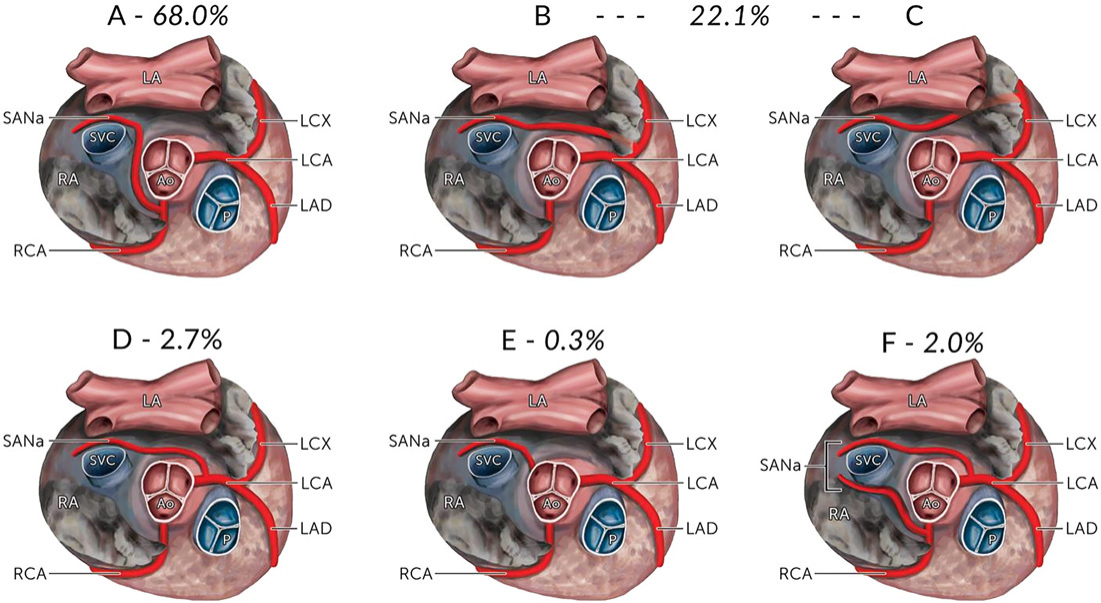
The various origins of the sinoatrial nodal artery (SANa) as seen from a superior view with their calculated pooled prevalence in the overall population. (A) From the Right Coronary Artery; (B) From the Left Circumflex Artery (proximal); (C) From the Left Circumflex Artery (distal); (D) From the Left Coronary Artery; (E) From the Aorta; (F) Dual origin from the Right Coronary Artery and the Left Circumflex Artery. The prevalence rates for B and C are reported as a combined rate. LA, Left Atrium; RA, Right Atrium; SVC, Superior Vena Cava; Ao, Aorta; P, Pulmonary Trunk; RCA, Right Coronary Artery; LCA, Left Coronary Artery; LCX, Left Circumflex Artery; LAD, Left Anterior Descending Artery; SANa, Sinoatrial Nodal Artery.
