# Supplementary material for: Anatomical Variations in the Sinoatrial Nodal Artery: A Meta-Analysis and Clinical Considerations
Source: PLoS One. 2016 Feb 5;11(2):e0148331. doi: 10.1371/journal.pone.0148331 (PMC4743947; doi:10.1371/journal.pone.0148331)
Supplement: S1 Fig — (PDF) [file pone.0148331.s002.pdf]

Number of the sinoatrial nodal artery (SANa) in the general population.

Single SANa

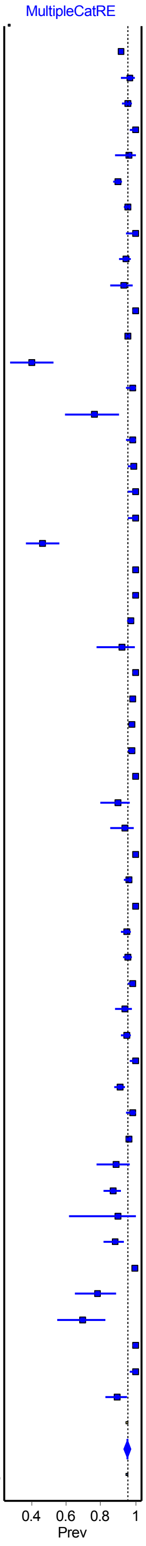

Study	Prev (95% CI)	% Weight
Andreini 2010	0.92 (0.90, 0.93)	2.16
Ballesteros 2010	0.97 (0.92, 1.00)	1.88
Ballesteros 2011	0.95 (0.92, 0.98)	2.05
Berdajs 2003	1.00 (0.97, 1.00)	1.71
Busquet 1984	0.96 (0.88, 1.00)	1.71
Cademartiri 2008	0.90 (0.87, 0.92)	2.11
Cezlan 2012	0.96 (0.94, 0.98)	2.10
Chen 1978	1.00 (0.94, 1.00)	1.49
Chi 2013	0.94 (0.91, 0.97)	2.03
Christides 1975	0.93 (0.85, 0.99)	1.77
DiDio 1995	1.00 (0.98, 1.00)	1.91
Dong 2013	0.95 (0.95, 0.96)	2.16
Eliska 1990	0.40 (0.28, 0.53)	1.77
Engel 1975	0.99 (0.95, 1.00)	1.85
Futami 2003	0.77 (0.60, 0.90)	1.49
Hromada 1969	0.99 (0.95, 1.00)	1.85
Hromada 1970	0.99 (0.96, 1.00)	1.91
Hutchinson 1976	1.00 (0.96, 1.00)	1.62
James 1958	1.00 (0.96, 1.00)	1.61
Kawashima 2003	0.46 (0.37, 0.56)	1.92
Kosar 2009	1.00 (1.00, 1.00)	2.13
Krupa 1993	1.00 (0.98, 1.00)	1.94
Kyriakidis 1988	0.97 (0.95, 0.99)	2.08
Lienhard 1972	0.92 (0.77, 1.00)	1.41
Mandard 1968	1.00 (0.98, 1.00)	1.91
Nerantzis 1980	0.99 (0.97, 1.00)	2.08
Nerantzis 2010	0.98 (0.96, 0.99)	2.10
Nerantzis 2011	0.98 (0.95, 1.00)	2.03
Okmen 2009	1.00 (1.00, 1.00)	2.15
Onciu 2006	0.90 (0.80, 0.97)	1.71
Ortale 2006	0.94 (0.85, 0.99)	1.71
Ou 2010	1.00 (1.00, 1.00)	2.16
Ozturk 2010	0.96 (0.93, 0.98)	2.06
Pejkovic 2008	1.00 (0.99, 1.00)	1.99
Peng 2009	0.95 (0.92, 0.97)	2.07
Ramanathan 2009	0.96 (0.93, 0.98)	2.08
Romhilt 1968	0.98 (0.96, 1.00)	2.02
Saremi 2008 (arterial supply..)	0.94 (0.88, 0.98)	1.91
Saremi 2008 (MDCT..)	0.95 (0.92, 0.97)	2.06
Shi 1983	1.00 (0.97, 1.00)	1.71
Shimotakahara 2014	0.91 (0.88, 0.94)	2.08
Song 2002	0.99 (0.95, 1.00)	1.85
Song 2012	0.96 (0.95, 0.98)	2.11
Sow 1996	0.89 (0.78, 0.97)	1.67
Tian 2012	0.87 (0.81, 0.92)	2.00
Verhaeghe 1967	0.90 (0.62, 1.00)	0.94
Vieweg 1975	0.88 (0.82, 0.93)	1.95
Wei 1987	1.00 (0.99, 1.00)	2.15
Xiao 1984	0.78 (0.65, 0.89)	1.67
Yangni-Angate 1995	0.70 (0.55, 0.83)	1.65
Yildirim 2010	1.00 (0.98, 1.00)	1.93
Yu 1980	1.00 (0.97, 1.00)	1.71
Zhang 2008	0.90 (0.83, 0.95)	1.90
Overall	0.95 (0.94, 0.97)	100.00
Q=1227.78, p=0.00, I2=96%		

Double SANa

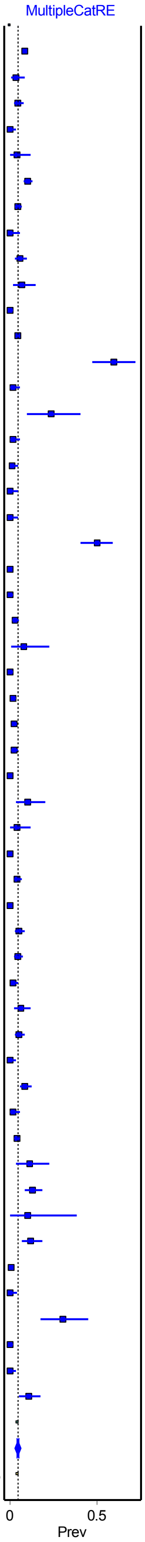

Study	Prev (95% CI)	% Weight
Andreini 2010	0.08 (0.07, 0.10)	2.16
Ballesteros 2010	0.03 (0.00, 0.08)	1.88
Ballesteros 2011	0.05 (0.02, 0.08)	2.05
Berdajs 2003	0.00 (0.00, 0.03)	1.71
Busquet 1984	0.04 (0.00, 0.12)	1.71
Cademartiri 2008	0.10 (0.08, 0.13)	2.11
Cezlan 2012	0.04 (0.02, 0.06)	2.10
Chen 1978	0.00 (0.00, 0.06)	1.49
Chi 2013	0.06 (0.03, 0.09)	2.03
Christides 1975	0.07 (0.01, 0.15)	1.77
DiDio 1995	0.00 (0.00, 0.02)	1.91
Dong 2013	0.05 (0.04, 0.05)	2.16
Eliska 1990	0.60 (0.47, 0.72)	1.77
Engel 1975	0.01 (0.00, 0.05)	1.85
Futami 2003	0.23 (0.10, 0.40)	1.49
Hromada 1969	0.01 (0.00, 0.05)	1.85
Hromada 1970	0.01 (0.00, 0.04)	1.91
Hutchinson 1976	0.00 (0.00, 0.04)	1.62
James 1958	0.00 (0.00, 0.04)	1.61
Kawashima 2003	0.50 (0.40, 0.60)	1.92
Kosar 2009	0.00 (0.00, 0.00)	2.13
Krupa 1993	0.00 (0.00, 0.02)	1.94
Kyriakidis 1988	0.03 (0.01, 0.05)	2.08
Lienhard 1972	0.08 (0.00, 0.23)	1.41
Mandard 1968	0.00 (0.00, 0.02)	1.91
Nerantzis 1980	0.01 (0.00, 0.03)	2.08
Nerantzis 2010	0.02 (0.01, 0.04)	2.10
Nerantzis 2011	0.02 (0.00, 0.05)	2.03
Okmen 2009	0.00 (0.00, 0.00)	2.15
Onciu 2006	0.10 (0.03, 0.20)	1.71
Ortale 2006	0.04 (0.00, 0.12)	1.71
Ou 2010	0.00 (0.00, 0.00)	2.16
Ozturk 2010	0.04 (0.02, 0.07)	2.06
Pejkovic 2008	0.00 (0.00, 0.01)	1.99
Peng 2009	0.05 (0.03, 0.08)	2.07
Ramanathan 2009	0.04 (0.02, 0.07)	2.08
Romhilt 1968	0.02 (0.00, 0.04)	2.02
Saremi 2008 (arterial supply..)	0.06 (0.02, 0.12)	1.91
Saremi 2008 (MDCT..)	0.05 (0.03, 0.08)	2.06
Shi 1983	0.00 (0.00, 0.03)	1.71
Shimotakahara 2014	0.09 (0.06, 0.12)	2.08
Song 2002	0.01 (0.00, 0.05)	1.85
Song 2012	0.04 (0.02, 0.05)	2.11
Sow 1996	0.11 (0.03, 0.22)	1.67
Tian 2012	0.13 (0.08, 0.19)	2.00
Verhaeghe 1967	0.10 (0.00, 0.38)	0.94
Vieweg 1975	0.12 (0.07, 0.18)	1.95
Wei 1987	0.00 (0.00, 0.01)	2.15
Xiao 1984	0.00 (0.00, 0.04)	1.67
Yangni-Angate 1995	0.30 (0.17, 0.45)	1.65
Yildirim 2010	0.00 (0.00, 0.02)	1.93
Yu 1980	0.00 (0.00, 0.03)	1.71
Zhang 2008	0.10 (0.05, 0.17)	1.90
Overall	0.04 (0.03, 0.06)	100.00

Q=1227.78, p=0.00, I2=96%

Triple SANa

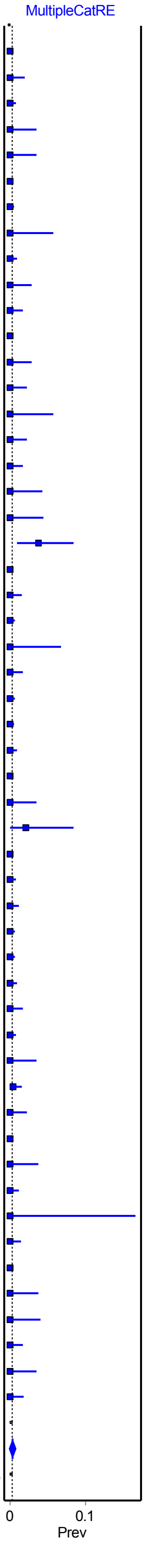

Study	Prev (95% CI)	% Weight
Andreini 2010	0.00 (0.00, 0.00)	2.16
Ballesteros 2010	0.00 (0.00, 0.02)	1.88
Ballesteros 2011	0.00 (0.00, 0.01)	2.05
Berdajs 2003	0.00 (0.00, 0.03)	1.71
Busquet 1984	0.00 (0.00, 0.03)	1.71
Cademartiri 2008	0.00 (0.00, 0.00)	2.11
Cezlan 2012	0.00 (0.00, 0.00)	2.10
Chen 1978	0.00 (0.00, 0.06)	1.49
Chi 2013	0.00 (0.00, 0.01)	2.03
Christides 1975	0.00 (0.00, 0.03)	1.77
DiDio 1995	0.00 (0.00, 0.02)	1.91
Dong 2013	0.00 (0.00, 0.00)	2.16
Eliska 1990	0.00 (0.00, 0.03)	1.77
Engel 1975	0.00 (0.00, 0.02)	1.85
Futami 2003	0.00 (0.00, 0.06)	1.49
Hromada 1969	0.00 (0.00, 0.02)	1.85
Hromada 1970	0.00 (0.00, 0.02)	1.91
Hutchinson 1976	0.00 (0.00, 0.04)	1.62
James 1958	0.00 (0.00, 0.04)	1.61
Kawashima 2003	0.04 (0.01, 0.08)	1.92
Kosar 2009	0.00 (0.00, 0.00)	2.13
Krupa 1993	0.00 (0.00, 0.02)	1.94
Kyriakidis 1988	0.00 (0.00, 0.01)	2.08
Lienhard 1972	0.00 (0.00, 0.07)	1.41
Mandard 1968	0.00 (0.00, 0.02)	1.91
Nerantzis 1980	0.00 (0.00, 0.01)	2.08
Nerantzis 2010	0.00 (0.00, 0.00)	2.10
Nerantzis 2011	0.00 (0.00, 0.01)	2.03
Okmen 2009	0.00 (0.00, 0.00)	2.15
Onciu 2006	0.00 (0.00, 0.03)	1.71
Ortale 2006	0.02 (0.00, 0.08)	1.71
Ou 2010	0.00 (0.00, 0.00)	2.16
Ozturk 2010	0.00 (0.00, 0.01)	2.06
Pejkovic 2008	0.00 (0.00, 0.01)	1.99
Peng 2009	0.00 (0.00, 0.01)	2.07
Ramanathan 2009	0.00 (0.00, 0.01)	2.08
Romhilt 1968	0.00 (0.00, 0.01)	2.02
Saremi 2008 (arterial supply..)	0.00 (0.00, 0.02)	1.91
Saremi 2008 (MDCT..)	0.00 (0.00, 0.01)	2.06
Shi 1983	0.00 (0.00, 0.03)	1.71
Shimotakahara 2014	0.00 (0.00, 0.01)	2.08
Song 2002	0.00 (0.00, 0.02)	1.85
Song 2012	0.00 (0.00, 0.00)	2.11
Sow 1996	0.00 (0.00, 0.04)	1.67
Tian 2012	0.00 (0.00, 0.01)	2.00
Verhaeghe 1967	0.00 (0.00, 0.17)	0.94
Vieweg 1975	0.00 (0.00, 0.01)	1.95
Wei 1987	0.00 (0.00, 0.00)	2.15
Xiao 1984	0.00 (0.00, 0.04)	1.67
Yangni-Angate 1995	0.00 (0.00, 0.04)	1.65
Yildirim 2010	0.00 (0.00, 0.02)	1.93
Yu 1980	0.00 (0.00, 0.03)	1.71
Zhang 2008	0.00 (0.00, 0.02)	1.90
Overall	0.00 (0.00, 0.01)	100.00

Q=1227.78, p=0.00, I2=96%
