# Supplementary material for: Anatomical Variations in the Sinoatrial Nodal Artery: A Meta-Analysis and Clinical Considerations
Source: PLoS One. 2016 Feb 5;11(2):e0148331. doi: 10.1371/journal.pone.0148331 (PMC4743947; doi:10.1371/journal.pone.0148331)
Supplement: S2 Fig — (PDF) [file pone.0148331.s003.pdf]

Origin of the sinoatrial nodal artery (SANa)

SANa arising from RCA

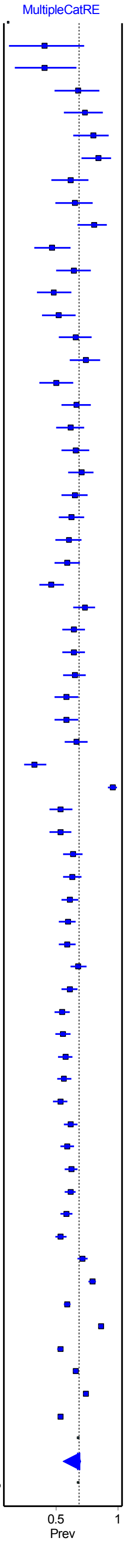

Study	Prev (95% CI)	% Weight
Verhaeghe 1967	0.40 (0.11, 0.72)	1.27
Ndiaye1994	0.40 (0.16, 0.66)	1.38
Lienhard 1972	0.68 (0.48, 0.85)	1.48
Futami 2003	0.73 (0.56, 0.88)	1.51
Chen 1978	0.80 (0.64, 0.93)	1.51
Bokeriia 2014	0.84 (0.71, 0.94)	1.54
James 1958	0.62 (0.46, 0.76)	1.55
Hutchinson 1976	0.65 (0.49, 0.79)	1.55
Xiao 1984	0.80 (0.67, 0.91)	1.55
Yangni-Angate 1995	0.47 (0.32, 0.62)	1.56
Sow 1996	0.64 (0.50, 0.78)	1.56
Shi 1983	0.48 (0.34, 0.62)	1.57
Yu 1980	0.52 (0.38, 0.66)	1.57
Busquet 1984	0.66 (0.52, 0.79)	1.57
Onciu 2006	0.74 (0.61, 0.85)	1.57
Ortale 2006	0.50 (0.36, 0.64)	1.57
Christides 1975	0.67 (0.54, 0.78)	1.59
Bokeriya 1984	0.61 (0.50, 0.73)	1.60
Kennel 1972	0.66 (0.54, 0.76)	1.60
Yokokawa 2012	0.70 (0.59, 0.80)	1.61
Song 2002	0.65 (0.54, 0.75)	1.61
Hromada 1969	0.63 (0.52, 0.73)	1.61
Engel 1975	0.60 (0.49, 0.71)	1.61
Ballesteros 2010	0.59 (0.49, 0.69)	1.62
Zhang 2008	0.46 (0.36, 0.56)	1.62
Sahni 1988	0.73 (0.64, 0.81)	1.63
Voegtlin 1968	0.64 (0.54, 0.73)	1.63
Mandard 1968	0.64 (0.54, 0.73)	1.63
Hromada 1970	0.65 (0.55, 0.74)	1.63
DiDio 1995	0.58 (0.48, 0.68)	1.63
Caetano 1995	0.58 (0.48, 0.68)	1.63
Saremi 2008 (arterial supply..)	0.66 (0.57, 0.75)	1.63
Kawashima 2003	0.32 (0.23, 0.41)	1.63
Yildirim 2010	0.96 (0.92, 0.99)	1.63
Krupa 1993	0.54 (0.44, 0.63)	1.63
Vieweg 1975	0.53 (0.44, 0.62)	1.63
Pejkovic 2008	0.63 (0.55, 0.71)	1.64
Tian 2012	0.63 (0.55, 0.71)	1.65
Romhilt 1968	0.61 (0.54, 0.68)	1.65
Chi 2013	0.59 (0.52, 0.66)	1.65
Nerantzis 2011	0.59 (0.52, 0.66)	1.65
Crainicianu 1921	0.68 (0.61, 0.74)	1.65
Ballesteros 2011	0.61 (0.54, 0.67)	1.66
Saremi 2008 (MDCT..)	0.55 (0.48, 0.61)	1.66
Ozturk 2010	0.55 (0.49, 0.61)	1.66
Peng 2009	0.57 (0.51, 0.63)	1.66
Shimotakahara 2014	0.56 (0.51, 0.62)	1.66
Ramanathan 2009	0.53 (0.47, 0.59)	1.66
Nerantzis 1980	0.62 (0.56, 0.67)	1.66
Kyriakidis 1988	0.59 (0.53, 0.64)	1.66
Nerantzis 1983	0.62 (0.57, 0.67)	1.67
Nerantzis 2010	0.61 (0.56, 0.66)	1.67
Cezlan 2012	0.58 (0.53, 0.63)	1.67
Song 2012	0.53 (0.49, 0.58)	1.67
Cademartiri 2008	0.71 (0.67, 0.75)	1.67
Kosar 2009	0.79 (0.76, 0.82)	1.67
Wei 1987	0.59 (0.56, 0.61)	1.68
Okmen 2009	0.86 (0.84, 0.88)	1.68
Ou 2010	0.53 (0.51, 0.55)	1.68
Erol 2012	0.66 (0.64, 0.68)	1.68
Andreini 2010	0.74 (0.72, 0.75)	1.68
Dong 2013	0.53 (0.51, 0.55)	1.68
Overall	0.68 (0.56, 0.69)	100.00

Q=5758.96, p=0.00, I2=99%

SANa arising from LCX

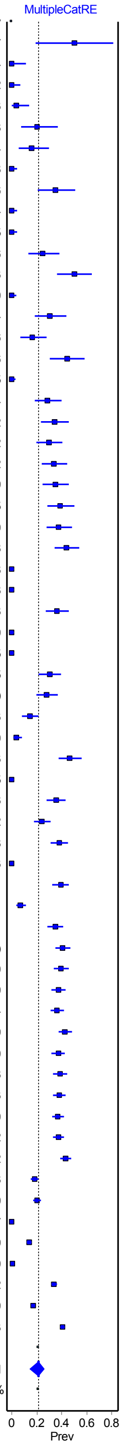

Study	Prev (95% CI)	% Weight
Verhaeghe 1967	0.50 (0.19, 0.81)	1.27
Ndiaye1994	0.00 (0.00, 0.11)	1.38
Lienhard 1972	0.00 (0.00, 0.07)	1.48
Futami 2003	0.03 (0.00, 0.14)	1.51
Chen 1978	0.20 (0.07, 0.36)	1.51
Bokeriia 2014	0.16 (0.06, 0.29)	1.54
James 1958	0.00 (0.00, 0.04)	1.55
Hutchinson 1976	0.35 (0.21, 0.51)	1.55
Xiao 1984	0.00 (0.00, 0.04)	1.55
Yangni-Angate 1995	0.00 (0.00, 0.04)	1.56
Sow 1996	0.24 (0.13, 0.38)	1.56
Shi 1983	0.50 (0.36, 0.64)	1.57
Yu 1980	0.00 (0.00, 0.03)	1.57
Busquet 1984	0.30 (0.18, 0.44)	1.57
Onciu 2006	0.16 (0.07, 0.28)	1.57
Ortale 2006	0.44 (0.30, 0.58)	1.57
Christides 1975	0.00 (0.00, 0.03)	1.59
Bokeriya 1984	0.29 (0.19, 0.40)	1.60
Kennel 1972	0.34 (0.24, 0.46)	1.60
Yokokawa 2012	0.30 (0.20, 0.41)	1.61
Song 2002	0.34 (0.24, 0.45)	1.61
Hromada 1969	0.35 (0.25, 0.46)	1.61
Engel 1975	0.39 (0.28, 0.50)	1.61
Ballesteros 2010	0.38 (0.28, 0.48)	1.62
Zhang 2008	0.44 (0.34, 0.54)	1.62
Sahni 1988	0.00 (0.00, 0.02)	1.63
Voegtlin 1968	0.00 (0.00, 0.02)	1.63
Mandard 1968	0.36 (0.27, 0.46)	1.63
Hromada 1970	0.00 (0.00, 0.02)	1.63
DiDio 1995	0.00 (0.00, 0.02)	1.63
Caetano 1995	0.30 (0.21, 0.39)	1.63
Saremi 2008 (arterial supply..)	0.28 (0.19, 0.37)	1.63
Kawashima 2003	0.14 (0.08, 0.22)	1.63
Yildirim 2010	0.04 (0.01, 0.08)	1.63
Krupa 1993	0.46 (0.37, 0.56)	1.63
Vieweg 1975	0.00 (0.00, 0.01)	1.63
Pejkovic 2008	0.35 (0.28, 0.43)	1.64
Tian 2012	0.24 (0.17, 0.31)	1.65
Romhilt 1968	0.38 (0.31, 0.45)	1.65
Chi 2013	0.00 (0.00, 0.01)	1.65
Nerantzis 2011	0.39 (0.32, 0.46)	1.65
Crainicianu 1921	0.07 (0.04, 0.11)	1.65
Ballesteros 2011	0.35 (0.29, 0.41)	1.66
Saremi 2008 (MDCT..)	0.41 (0.34, 0.47)	1.66
Ozturk 2010	0.39 (0.33, 0.46)	1.66
Peng 2009	0.38 (0.32, 0.43)	1.66
Shimotakahara 2014	0.36 (0.31, 0.42)	1.66
Ramanathan 2009	0.43 (0.37, 0.48)	1.66
Nerantzis 1980	0.37 (0.32, 0.43)	1.66
Kyriakidis 1988	0.39 (0.33, 0.44)	1.66
Nerantzis 1983	0.38 (0.33, 0.43)	1.67
Nerantzis 2010	0.37 (0.32, 0.42)	1.67
Cezlan 2012	0.37 (0.33, 0.42)	1.67
Song 2012	0.43 (0.39, 0.47)	1.67
Cademartiri 2008	0.18 (0.15, 0.22)	1.67
Kosar 2009	0.20 (0.17, 0.23)	1.67
Wei 1987	0.00 (0.00, 0.00)	1.68
Okmen 2009	0.14 (0.12, 0.16)	1.68
Ou 2010	0.01 (0.00, 0.01)	1.68
Erol 2012	0.34 (0.32, 0.36)	1.68
Andreini 2010	0.17 (0.15, 0.18)	1.68
Dong 2013	0.41 (0.39, 0.42)	1.68
Overall	0.22 (0.15, 0.26)	100.00

Q=5758.96, p=0.00, I2=99%

SANa arising from LCA

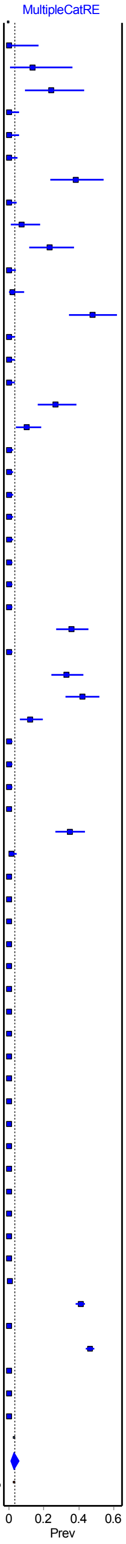

Study	Prev (95% CI)	% Weight
Verhaeghe 1967	0.00 (0.00, 0.17)	1.27
Ndiaye1994	0.13 (0.00, 0.36)	1.38
Lienhard 1972	0.24 (0.09, 0.43)	1.48
Futami 2003	0.00 (0.00, 0.06)	1.51
Chen 1978	0.00 (0.00, 0.06)	1.51
Bokeriia 2014	0.00 (0.00, 0.04)	1.54
James 1958	0.38 (0.24, 0.54)	1.55
Hutchinson 1976	0.00 (0.00, 0.04)	1.55
Xiao 1984	0.07 (0.01, 0.18)	1.55
Yangni-Angate 1995	0.23 (0.12, 0.37)	1.56
Sow 1996	0.00 (0.00, 0.04)	1.56
Shi 1983	0.02 (0.00, 0.08)	1.57
Yu 1980	0.48 (0.34, 0.62)	1.57
Busquet 1984	0.00 (0.00, 0.03)	1.57
Onciu 2006	0.00 (0.00, 0.03)	1.57
Ortale 2006	0.00 (0.00, 0.03)	1.57
Christides 1975	0.27 (0.16, 0.39)	1.59
Bokeriya 1984	0.10 (0.04, 0.18)	1.60
Kennel 1972	0.00 (0.00, 0.02)	1.60
Yokokawa 2012	0.00 (0.00, 0.02)	1.61
Song 2002	0.00 (0.00, 0.02)	1.61
Hromada 1969	0.00 (0.00, 0.02)	1.61
Engel 1975	0.00 (0.00, 0.02)	1.61
Ballesteros 2010	0.00 (0.00, 0.02)	1.62
Zhang 2008	0.00 (0.00, 0.02)	1.62
Sahni 1988	0.00 (0.00, 0.02)	1.63
Voegtlin 1968	0.36 (0.27, 0.46)	1.63
Mandard 1968	0.00 (0.00, 0.02)	1.63
Hromada 1970	0.33 (0.24, 0.43)	1.63
DiDio 1995	0.42 (0.32, 0.52)	1.63
Caetano 1995	0.12 (0.06, 0.19)	1.63
Saremi 2008 (arterial supply..)	0.00 (0.00, 0.02)	1.63
Kawashima 2003	0.00 (0.00, 0.02)	1.63
Yildirim 2010	0.00 (0.00, 0.02)	1.63
Krupa 1993	0.00 (0.00, 0.02)	1.63
Vieweg 1975	0.35 (0.26, 0.44)	1.63
Pejkovic 2008	0.01 (0.00, 0.04)	1.64
Tian 2012	0.00 (0.00, 0.01)	1.65
Romhilt 1968	0.00 (0.00, 0.01)	1.65
Chi 2013	0.00 (0.00, 0.01)	1.65
Nerantzis 2011	0.00 (0.00, 0.01)	1.65
Crainicianu 1921	0.00 (0.00, 0.01)	1.65
Ballesteros 2011	0.00 (0.00, 0.01)	1.66
Saremi 2008 (MDCT..)	0.00 (0.00, 0.01)	1.66
Ozturk 2010	0.00 (0.00, 0.01)	1.66
Peng 2009	0.00 (0.00, 0.01)	1.66
Shimotakahara 2014	0.00 (0.00, 0.01)	1.66
Ramanathan 2009	0.00 (0.00, 0.01)	1.66
Nerantzis 1980	0.00 (0.00, 0.01)	1.66
Kyriakidis 1988	0.00 (0.00, 0.01)	1.66
Nerantzis 1983	0.00 (0.00, 0.00)	1.67
Nerantzis 2010	0.00 (0.00, 0.00)	1.67
Cezlan 2012	0.00 (0.00, 0.00)	1.67
Song 2012	0.00 (0.00, 0.00)	1.67
Cademartiri 2008	0.00 (0.00, 0.00)	1.67
Kosar 2009	0.00 (0.00, 0.01)	1.67
Wei 1987	0.41 (0.38, 0.44)	1.68
Okmen 2009	0.00 (0.00, 0.00)	1.68
Ou 2010	0.47 (0.44, 0.49)	1.68
Erol 2012	0.00 (0.00, 0.00)	1.68
Andreini 2010	0.00 (0.00, 0.00)	1.68
Dong 2013	0.00 (0.00, 0.00)	1.68
Overall	0.03 (0.01, 0.06)	100.00

Q=5758.96, p=0.00, I2=99%

SANa arising from aorta

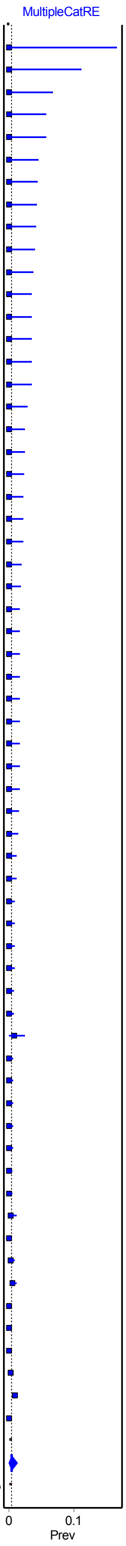

Study	Prev (95% CI)	% Weight
Verhaeghe 1967	0.00 (0.00, 0.17)	1.27
Ndiaye1994	0.00 (0.00, 0.11)	1.38
Lienhard 1972	0.00 (0.00, 0.07)	1.48
Futami 2003	0.00 (0.00, 0.06)	1.51
Chen 1978	0.00 (0.00, 0.06)	1.51
Bokeriia 2014	0.00 (0.00, 0.04)	1.54
James 1958	0.00 (0.00, 0.04)	1.55
Hutchinson 1976	0.00 (0.00, 0.04)	1.55
Xiao 1984	0.00 (0.00, 0.04)	1.55
Yangni-Angate 1995	0.00 (0.00, 0.04)	1.56
Sow 1996	0.00 (0.00, 0.04)	1.56
Shi 1983	0.00 (0.00, 0.03)	1.57
Yu 1980	0.00 (0.00, 0.03)	1.57
Busquet 1984	0.00 (0.00, 0.03)	1.57
Onciu 2006	0.00 (0.00, 0.03)	1.57
Ortale 2006	0.00 (0.00, 0.03)	1.57
Christides 1975	0.00 (0.00, 0.03)	1.59
Bokeriya 1984	0.00 (0.00, 0.02)	1.60
Kennel 1972	0.00 (0.00, 0.02)	1.60
Yokokawa 2012	0.00 (0.00, 0.02)	1.61
Song 2002	0.00 (0.00, 0.02)	1.61
Hromada 1969	0.00 (0.00, 0.02)	1.61
Engel 1975	0.00 (0.00, 0.02)	1.61
Ballesteros 2010	0.00 (0.00, 0.02)	1.62
Zhang 2008	0.00 (0.00, 0.02)	1.62
Sahni 1988	0.00 (0.00, 0.02)	1.63
Voegtlin 1968	0.00 (0.00, 0.02)	1.63
Mandard 1968	0.00 (0.00, 0.02)	1.63
Hromada 1970	0.00 (0.00, 0.02)	1.63
DiDio 1995	0.00 (0.00, 0.02)	1.63
Caetano 1995	0.00 (0.00, 0.02)	1.63
Saremi 2008 (arterial supply..)	0.00 (0.00, 0.02)	1.63
Kawashima 2003	0.00 (0.00, 0.02)	1.63
Yildirim 2010	0.00 (0.00, 0.02)	1.63
Krupa 1993	0.00 (0.00, 0.02)	1.63
Vieweg 1975	0.00 (0.00, 0.01)	1.63
Pejkovic 2008	0.00 (0.00, 0.01)	1.64
Tian 2012	0.00 (0.00, 0.01)	1.65
Romhilt 1968	0.00 (0.00, 0.01)	1.65
Chi 2013	0.00 (0.00, 0.01)	1.65
Nerantzis 2011	0.00 (0.00, 0.01)	1.65
Crainicianu 1921	0.00 (0.00, 0.01)	1.65
Ballesteros 2011	0.00 (0.00, 0.01)	1.66
Saremi 2008 (MDCT..)	0.00 (0.00, 0.01)	1.66
Ozturk 2010	0.01 (0.00, 0.02)	1.66
Peng 2009	0.00 (0.00, 0.01)	1.66
Shimotakahara 2014	0.00 (0.00, 0.01)	1.66
Ramanathan 2009	0.00 (0.00, 0.01)	1.66
Nerantzis 1980	0.00 (0.00, 0.01)	1.66
Kyriakidis 1988	0.00 (0.00, 0.01)	1.66
Nerantzis 1983	0.00 (0.00, 0.00)	1.67
Nerantzis 2010	0.00 (0.00, 0.00)	1.67
Cezlan 2012	0.00 (0.00, 0.01)	1.67
Song 2012	0.00 (0.00, 0.00)	1.67
Cademartiri 2008	0.00 (0.00, 0.01)	1.67
Kosar 2009	0.00 (0.00, 0.01)	1.67
Wei 1987	0.00 (0.00, 0.00)	1.68
Okmen 2009	0.00 (0.00, 0.00)	1.68
Ou 2010	0.00 (0.00, 0.00)	1.68
Erol 2012	0.00 (0.00, 0.00)	1.68
Andreini 2010	0.01 (0.01, 0.01)	1.68
Dong 2013	0.00 (0.00, 0.00)	1.68
Overall	0.00 (0.00, 0.01)	100.00

Q=5758.96, p=0.00, I2=99%

SANa arising from bronchial artery

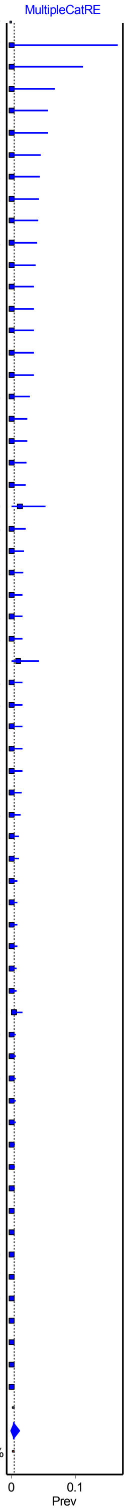

Study	Prev (95% CI)	% Weight
Verhaeghe 1967	0.00 (0.00, 0.17)	1.27
Ndiaye1994	0.00 (0.00, 0.11)	1.38
Lienhard 1972	0.00 (0.00, 0.07)	1.48
Futami 2003	0.00 (0.00, 0.06)	1.51
Chen 1978	0.00 (0.00, 0.06)	1.51
Bokeriia 2014	0.00 (0.00, 0.04)	1.54
James 1958	0.00 (0.00, 0.04)	1.55
Hutchinson 1976	0.00 (0.00, 0.04)	1.55
Xiao 1984	0.00 (0.00, 0.04)	1.55
Yangni-Angate 1995	0.00 (0.00, 0.04)	1.56
Sow 1996	0.00 (0.00, 0.04)	1.56
Shi 1983	0.00 (0.00, 0.03)	1.57
Yu 1980	0.00 (0.00, 0.03)	1.57
Busquet 1984	0.00 (0.00, 0.03)	1.57
Onciu 2006	0.00 (0.00, 0.03)	1.57
Ortale 2006	0.00 (0.00, 0.03)	1.57
Christides 1975	0.00 (0.00, 0.03)	1.59
Bokeriya 1984	0.00 (0.00, 0.02)	1.60
Kennel 1972	0.00 (0.00, 0.02)	1.60
Yokokawa 2012	0.00 (0.00, 0.02)	1.61
Song 2002	0.00 (0.00, 0.02)	1.61
Hromada 1969	0.01 (0.00, 0.05)	1.61
Engel 1975	0.00 (0.00, 0.02)	1.61
Ballesteros 2010	0.00 (0.00, 0.02)	1.62
Zhang 2008	0.00 (0.00, 0.02)	1.62
Sahni 1988	0.00 (0.00, 0.02)	1.63
Voegtlin 1968	0.00 (0.00, 0.02)	1.63
Mandard 1968	0.00 (0.00, 0.02)	1.63
Hromada 1970	0.01 (0.00, 0.04)	1.63
DiDio 1995	0.00 (0.00, 0.02)	1.63
Caetano 1995	0.00 (0.00, 0.02)	1.63
Saremi 2008 (arterial supply..)	0.00 (0.00, 0.02)	1.63
Kawashima 2003	0.00 (0.00, 0.02)	1.63
Yildirim 2010	0.00 (0.00, 0.02)	1.63
Krupa 1993	0.00 (0.00, 0.02)	1.63
Vieweg 1975	0.00 (0.00, 0.01)	1.63
Pejkovic 2008	0.00 (0.00, 0.01)	1.64
Tian 2012	0.00 (0.00, 0.01)	1.65
Romhilt 1968	0.00 (0.00, 0.01)	1.65
Chi 2013	0.00 (0.00, 0.01)	1.65
Nerantzis 2011	0.00 (0.00, 0.01)	1.65
Crainicianu 1921	0.00 (0.00, 0.01)	1.65
Ballesteros 2011	0.00 (0.00, 0.01)	1.66
Saremi 2008 (MDCT..)	0.00 (0.00, 0.01)	1.66
Ozturk 2010	0.00 (0.00, 0.02)	1.66
Peng 2009	0.00 (0.00, 0.01)	1.66
Shimotakahara 2014	0.00 (0.00, 0.01)	1.66
Ramanathan 2009	0.00 (0.00, 0.01)	1.66
Nerantzis 1980	0.00 (0.00, 0.01)	1.66
Kyriakidis 1988	0.00 (0.00, 0.01)	1.66
Nerantzis 1983	0.00 (0.00, 0.00)	1.67
Nerantzis 2010	0.00 (0.00, 0.00)	1.67
Cezlan 2012	0.00 (0.00, 0.00)	1.67
Song 2012	0.00 (0.00, 0.00)	1.67
Cademartiri 2008	0.00 (0.00, 0.00)	1.67
Kosar 2009	0.00 (0.00, 0.00)	1.67
Wei 1987	0.00 (0.00, 0.00)	1.68
Okmen 2009	0.00 (0.00, 0.00)	1.68
Ou 2010	0.00 (0.00, 0.00)	1.68
Erol 2012	0.00 (0.00, 0.00)	1.68
Andreini 2010	0.00 (0.00, 0.00)	1.68
Dong 2013	0.00 (0.00, 0.00)	1.68
Overall	0.00 (0.00, 0.01)	100.00

Q=5758.96, p=0.00, I2=99%

Duplicated SANa. One from RCA, the other from LCX

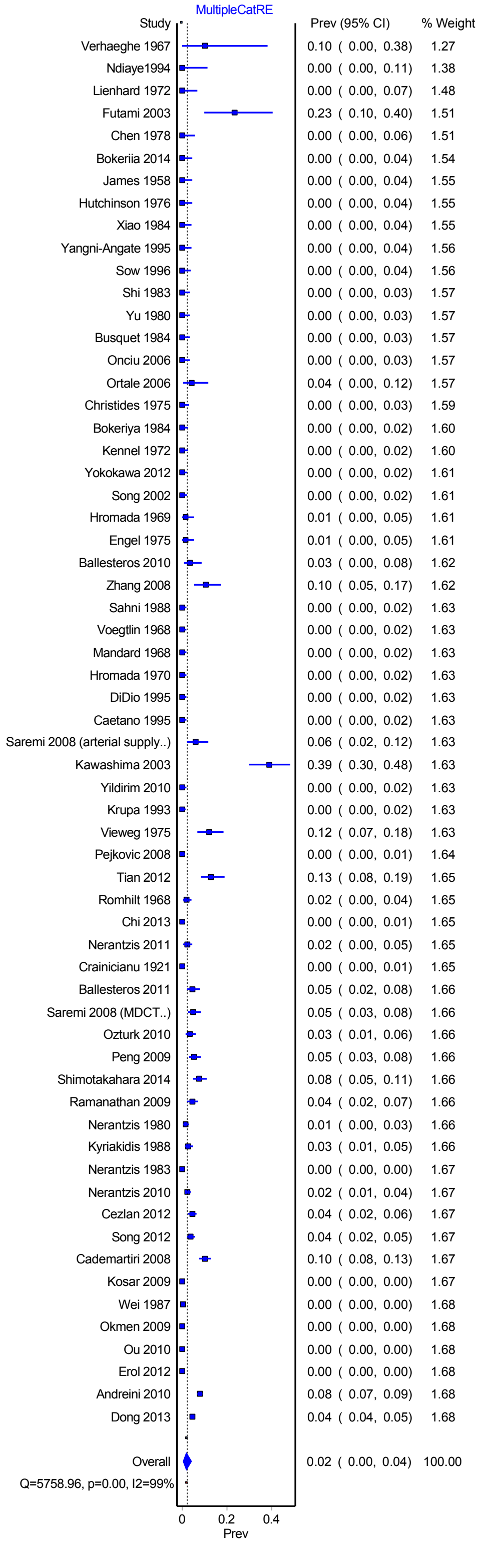

Duplicated SANa. One arising from RCA, the other arising from

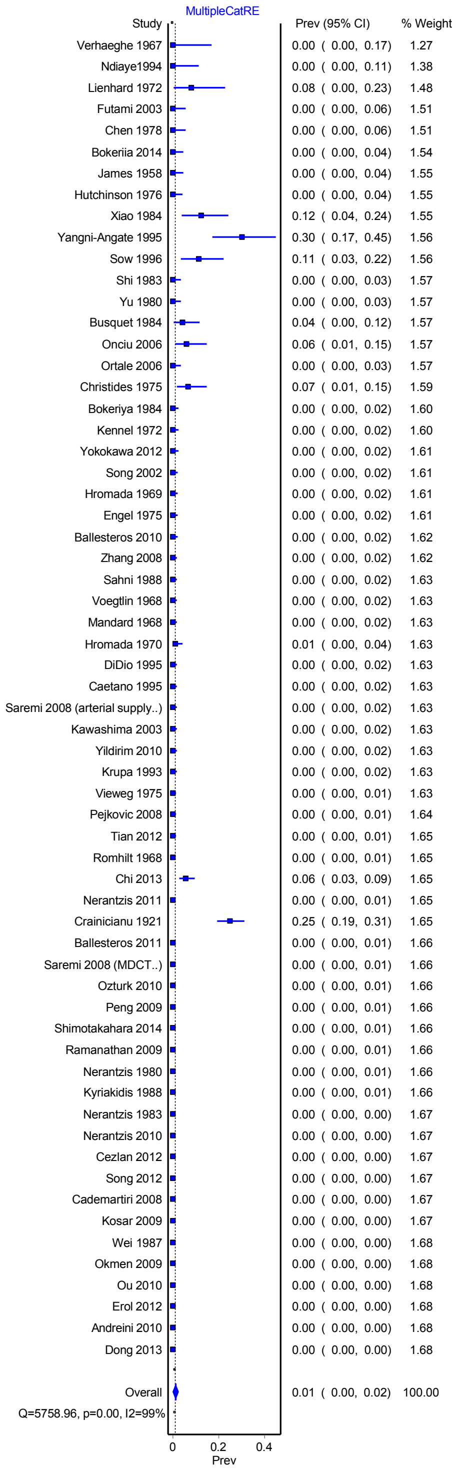

Duplicated SANa. One arising from RCA, the other arising from bronchial artery.

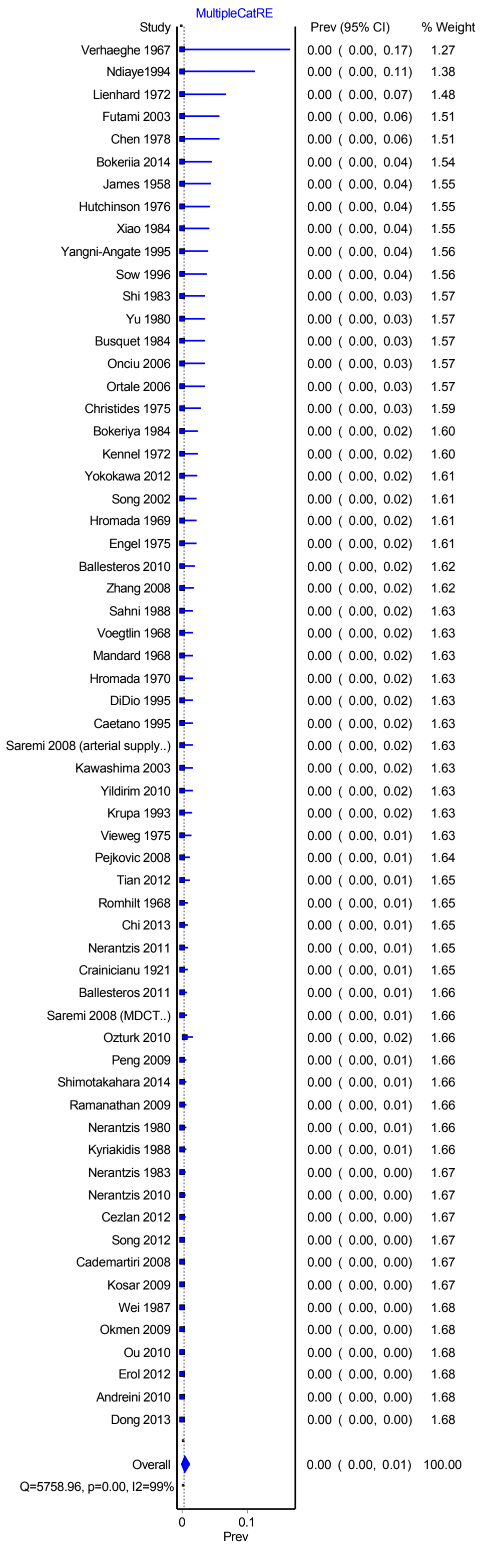

Duplicated SANa. One arising from LCX, the other arising from pulmonary artery

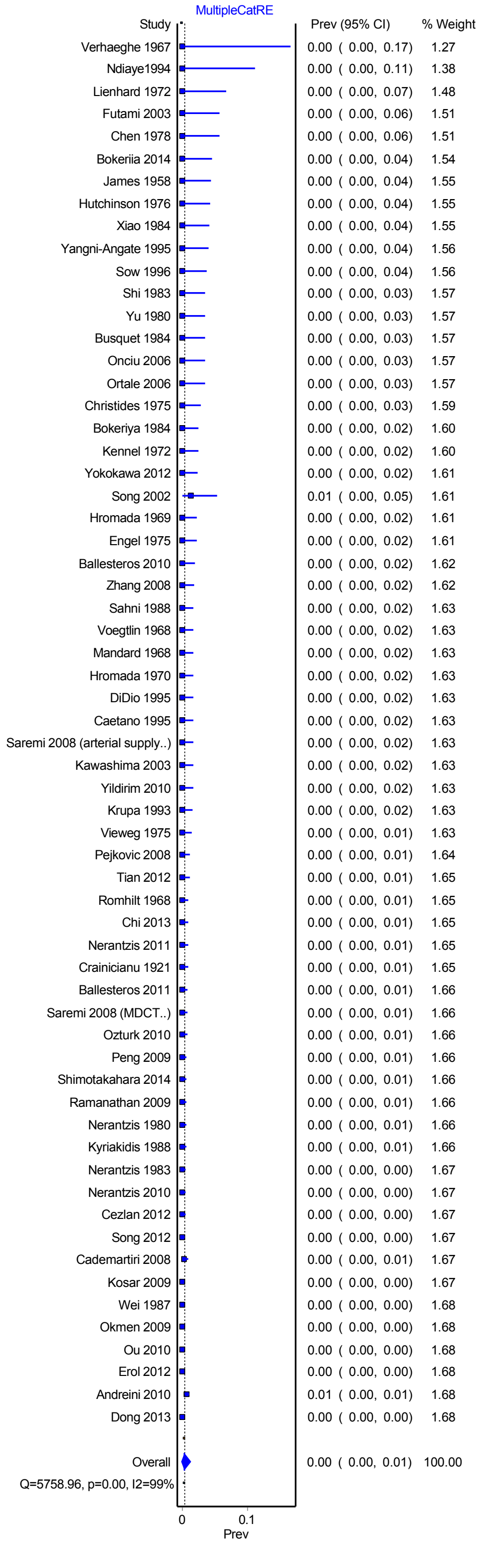

Duplicated SANa. One arising from LCX, the other arising from bronchial artery

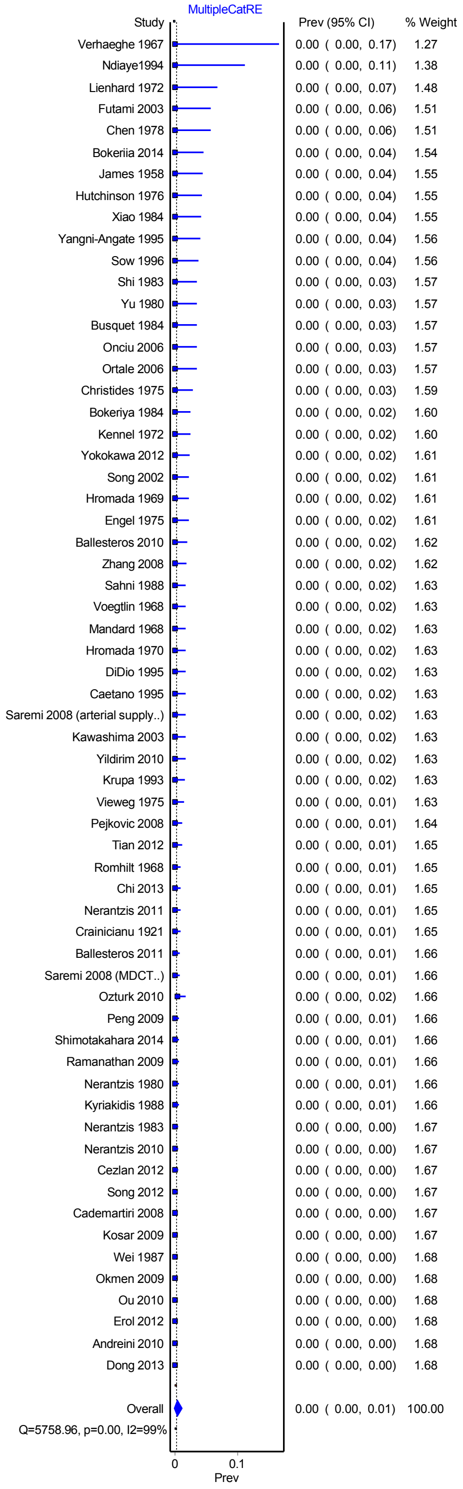

Duplicated SANa. Both arising from RCA.

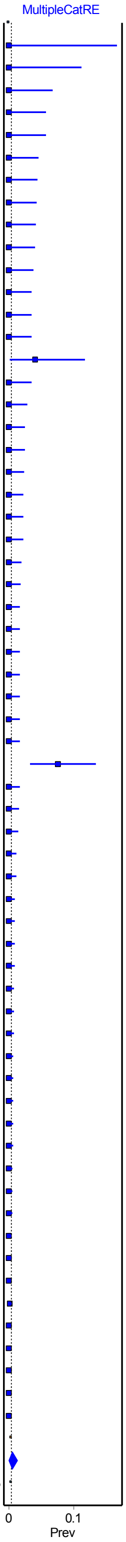

Study	Prev (95% CI)	% Weight
Verhaeghe 1967	0.00 (0.00, 0.17)	1.27
Ndiaye1994	0.00 (0.00, 0.11)	1.38
Lienhard 1972	0.00 (0.00, 0.07)	1.48
Futami 2003	0.00 (0.00, 0.06)	1.51
Chen 1978	0.00 (0.00, 0.06)	1.51
Bokeriia 2014	0.00 (0.00, 0.04)	1.54
James 1958	0.00 (0.00, 0.04)	1.55
Hutchinson 1976	0.00 (0.00, 0.04)	1.55
Xiao 1984	0.00 (0.00, 0.04)	1.55
Yangni-Angate 1995	0.00 (0.00, 0.04)	1.56
Sow 1996	0.00 (0.00, 0.04)	1.56
Shi 1983	0.00 (0.00, 0.03)	1.57
Yu 1980	0.00 (0.00, 0.03)	1.57
Busquet 1984	0.00 (0.00, 0.03)	1.57
Onciu 2006	0.04 (0.00, 0.12)	1.57
Ortale 2006	0.00 (0.00, 0.03)	1.57
Christides 1975	0.00 (0.00, 0.03)	1.59
Bokeriya 1984	0.00 (0.00, 0.02)	1.60
Kennel 1972	0.00 (0.00, 0.02)	1.60
Yokokawa 2012	0.00 (0.00, 0.02)	1.61
Song 2002	0.00 (0.00, 0.02)	1.61
Hromada 1969	0.00 (0.00, 0.02)	1.61
Engel 1975	0.00 (0.00, 0.02)	1.61
Ballesteros 2010	0.00 (0.00, 0.02)	1.62
Zhang 2008	0.00 (0.00, 0.02)	1.62
Sahni 1988	0.00 (0.00, 0.02)	1.63
Voegtlin 1968	0.00 (0.00, 0.02)	1.63
Mandard 1968	0.00 (0.00, 0.02)	1.63
Hromada 1970	0.00 (0.00, 0.02)	1.63
DiDio 1995	0.00 (0.00, 0.02)	1.63
Caetano 1995	0.00 (0.00, 0.02)	1.63
Saremi 2008 (arterial supply..)	0.00 (0.00, 0.02)	1.63
Kawashima 2003	0.08 (0.03, 0.13)	1.63
Yildirim 2010	0.00 (0.00, 0.02)	1.63
Krupa 1993	0.00 (0.00, 0.02)	1.63
Vieweg 1975	0.00 (0.00, 0.01)	1.63
Pejkovic 2008	0.00 (0.00, 0.01)	1.64
Tian 2012	0.00 (0.00, 0.01)	1.65
Romhilt 1968	0.00 (0.00, 0.01)	1.65
Chi 2013	0.00 (0.00, 0.01)	1.65
Nerantzis 2011	0.00 (0.00, 0.01)	1.65
Crainicianu 1921	0.00 (0.00, 0.01)	1.65
Ballesteros 2011	0.00 (0.00, 0.01)	1.66
Saremi 2008 (MDCT..)	0.00 (0.00, 0.01)	1.66
Ozturk 2010	0.00 (0.00, 0.01)	1.66
Peng 2009	0.00 (0.00, 0.01)	1.66
Shimotakahara 2014	0.00 (0.00, 0.01)	1.66
Ramanathan 2009	0.00 (0.00, 0.01)	1.66
Nerantzis 1980	0.00 (0.00, 0.01)	1.66
Kyriakidis 1988	0.00 (0.00, 0.01)	1.66
Nerantzis 1983	0.00 (0.00, 0.00)	1.67
Nerantzis 2010	0.00 (0.00, 0.00)	1.67
Cezlan 2012	0.00 (0.00, 0.00)	1.67
Song 2012	0.00 (0.00, 0.00)	1.67
Cademartiri 2008	0.00 (0.00, 0.00)	1.67
Kosar 2009	0.00 (0.00, 0.00)	1.67
Wei 1987	0.00 (0.00, 0.00)	1.68
Okmen 2009	0.00 (0.00, 0.00)	1.68
Ou 2010	0.00 (0.00, 0.00)	1.68
Erol 2012	0.00 (0.00, 0.00)	1.68
Andreini 2010	0.00 (0.00, 0.00)	1.68
Dong 2013	0.00 (0.00, 0.00)	1.68
Overall	0.00 (0.00, 0.01)	100.00
Q=5758.96, p=0.00, I2=99%		

Duplicated SANa. Both arising from LCX

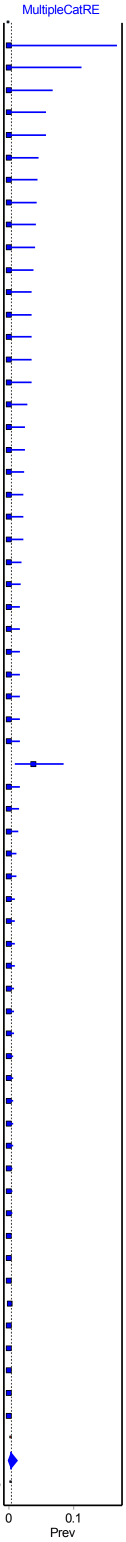

Study	Prev (95% CI)	% Weight
Verhaeghe 1967	0.00 (0.00, 0.17)	1.27
Ndiaye1994	0.00 (0.00, 0.11)	1.38
Lienhard 1972	0.00 (0.00, 0.07)	1.48
Futami 2003	0.00 (0.00, 0.06)	1.51
Chen 1978	0.00 (0.00, 0.06)	1.51
Bokeriia 2014	0.00 (0.00, 0.04)	1.54
James 1958	0.00 (0.00, 0.04)	1.55
Hutchinson 1976	0.00 (0.00, 0.04)	1.55
Xiao 1984	0.00 (0.00, 0.04)	1.55
Yangni-Angate 1995	0.00 (0.00, 0.04)	1.56
Sow 1996	0.00 (0.00, 0.04)	1.56
Shi 1983	0.00 (0.00, 0.03)	1.57
Yu 1980	0.00 (0.00, 0.03)	1.57
Busquet 1984	0.00 (0.00, 0.03)	1.57
Onciu 2006	0.00 (0.00, 0.03)	1.57
Ortale 2006	0.00 (0.00, 0.03)	1.57
Christides 1975	0.00 (0.00, 0.03)	1.59
Bokeriya 1984	0.00 (0.00, 0.02)	1.60
Kennel 1972	0.00 (0.00, 0.02)	1.60
Yokokawa 2012	0.00 (0.00, 0.02)	1.61
Song 2002	0.00 (0.00, 0.02)	1.61
Hromada 1969	0.00 (0.00, 0.02)	1.61
Engel 1975	0.00 (0.00, 0.02)	1.61
Ballesteros 2010	0.00 (0.00, 0.02)	1.62
Zhang 2008	0.00 (0.00, 0.02)	1.62
Sahni 1988	0.00 (0.00, 0.02)	1.63
Voegtlin 1968	0.00 (0.00, 0.02)	1.63
Mandard 1968	0.00 (0.00, 0.02)	1.63
Hromada 1970	0.00 (0.00, 0.02)	1.63
DiDio 1995	0.00 (0.00, 0.02)	1.63
Caetano 1995	0.00 (0.00, 0.02)	1.63
Saremi 2008 (arterial supply..)	0.00 (0.00, 0.02)	1.63
Kawashima 2003	0.04 (0.01, 0.08)	1.63
Yildirim 2010	0.00 (0.00, 0.02)	1.63
Krupa 1993	0.00 (0.00, 0.02)	1.63
Vieweg 1975	0.00 (0.00, 0.01)	1.63
Pejkovic 2008	0.00 (0.00, 0.01)	1.64
Tian 2012	0.00 (0.00, 0.01)	1.65
Romhilt 1968	0.00 (0.00, 0.01)	1.65
Chi 2013	0.00 (0.00, 0.01)	1.65
Nerantzis 2011	0.00 (0.00, 0.01)	1.65
Crainicianu 1921	0.00 (0.00, 0.01)	1.65
Ballesteros 2011	0.00 (0.00, 0.01)	1.66
Saremi 2008 (MDCT..)	0.00 (0.00, 0.01)	1.66
Ozturk 2010	0.00 (0.00, 0.01)	1.66
Peng 2009	0.00 (0.00, 0.01)	1.66
Shimotakahara 2014	0.00 (0.00, 0.01)	1.66
Ramanathan 2009	0.00 (0.00, 0.01)	1.66
Nerantzis 1980	0.00 (0.00, 0.01)	1.66
Kyriakidis 1988	0.00 (0.00, 0.01)	1.66
Nerantzis 1983	0.00 (0.00, 0.00)	1.67
Nerantzis 2010	0.00 (0.00, 0.00)	1.67
Cezlan 2012	0.00 (0.00, 0.00)	1.67
Song 2012	0.00 (0.00, 0.00)	1.67
Cademartiri 2008	0.00 (0.00, 0.00)	1.67
Kosar 2009	0.00 (0.00, 0.00)	1.67
Wei 1987	0.00 (0.00, 0.00)	1.68
Okmen 2009	0.00 (0.00, 0.00)	1.68
Ou 2010	0.00 (0.00, 0.00)	1.68
Erol 2012	0.00 (0.00, 0.00)	1.68
Andreini 2010	0.00 (0.00, 0.00)	1.68
Dong 2013	0.00 (0.00, 0.00)	1.68
Overall	0.00 (0.00, 0.01)	100.00

Q=5758.96, p=0.00, I2=99%

Duplicated SANa. Both arises from LCA

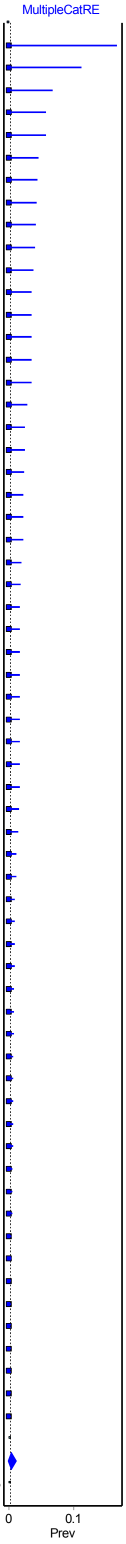

Study	Prev (95% CI)	% Weight
Verhaeghe 1967	0.00 (0.00, 0.17)	1.27
Ndiaye1994	0.00 (0.00, 0.11)	1.38
Lienhard 1972	0.00 (0.00, 0.07)	1.48
Futami 2003	0.00 (0.00, 0.06)	1.51
Chen 1978	0.00 (0.00, 0.06)	1.51
Bokeriia 2014	0.00 (0.00, 0.04)	1.54
James 1958	0.00 (0.00, 0.04)	1.55
Hutchinson 1976	0.00 (0.00, 0.04)	1.55
Xiao 1984	0.00 (0.00, 0.04)	1.55
Yangni-Angate 1995	0.00 (0.00, 0.04)	1.56
Sow 1996	0.00 (0.00, 0.04)	1.56
Shi 1983	0.00 (0.00, 0.03)	1.57
Yu 1980	0.00 (0.00, 0.03)	1.57
Busquet 1984	0.00 (0.00, 0.03)	1.57
Onciu 2006	0.00 (0.00, 0.03)	1.57
Ortale 2006	0.00 (0.00, 0.03)	1.57
Christides 1975	0.00 (0.00, 0.03)	1.59
Bokeriya 1984	0.00 (0.00, 0.02)	1.60
Kennel 1972	0.00 (0.00, 0.02)	1.60
Yokokawa 2012	0.00 (0.00, 0.02)	1.61
Song 2002	0.00 (0.00, 0.02)	1.61
Hromada 1969	0.00 (0.00, 0.02)	1.61
Engel 1975	0.00 (0.00, 0.02)	1.61
Ballesteros 2010	0.00 (0.00, 0.02)	1.62
Zhang 2008	0.00 (0.00, 0.02)	1.62
Sahni 1988	0.00 (0.00, 0.02)	1.63
Voegtlin 1968	0.00 (0.00, 0.02)	1.63
Mandard 1968	0.00 (0.00, 0.02)	1.63
Hromada 1970	0.00 (0.00, 0.02)	1.63
DiDio 1995	0.00 (0.00, 0.02)	1.63
Caetano 1995	0.00 (0.00, 0.02)	1.63
Saremi 2008 (arterial supply..)	0.00 (0.00, 0.02)	1.63
Kawashima 2003	0.00 (0.00, 0.02)	1.63
Yildirim 2010	0.00 (0.00, 0.02)	1.63
Krupa 1993	0.00 (0.00, 0.02)	1.63
Vieweg 1975	0.00 (0.00, 0.01)	1.63
Pejkovic 2008	0.00 (0.00, 0.01)	1.64
Tian 2012	0.00 (0.00, 0.01)	1.65
Romhilt 1968	0.00 (0.00, 0.01)	1.65
Chi 2013	0.00 (0.00, 0.01)	1.65
Nerantzis 2011	0.00 (0.00, 0.01)	1.65
Crainicianu 1921	0.00 (0.00, 0.01)	1.65
Ballesteros 2011	0.00 (0.00, 0.01)	1.66
Saremi 2008 (MDCT..)	0.00 (0.00, 0.01)	1.66
Ozturk 2010	0.00 (0.00, 0.01)	1.66
Peng 2009	0.00 (0.00, 0.01)	1.66
Shimotakahara 2014	0.00 (0.00, 0.01)	1.66
Ramanathan 2009	0.00 (0.00, 0.01)	1.66
Nerantzis 1980	0.00 (0.00, 0.01)	1.66
Kyriakidis 1988	0.00 (0.00, 0.01)	1.66
Nerantzis 1983	0.00 (0.00, 0.00)	1.67
Nerantzis 2010	0.00 (0.00, 0.00)	1.67
Cezlan 2012	0.00 (0.00, 0.00)	1.67
Song 2012	0.00 (0.00, 0.00)	1.67
Cademartiri 2008	0.00 (0.00, 0.00)	1.67
Kosar 2009	0.00 (0.00, 0.00)	1.67
Wei 1987	0.00 (0.00, 0.00)	1.68
Okmen 2009	0.00 (0.00, 0.00)	1.68
Ou 2010	0.00 (0.00, 0.00)	1.68
Erol 2012	0.00 (0.00, 0.00)	1.68
Andreini 2010	0.00 (0.00, 0.00)	1.68
Dong 2013	0.00 (0.00, 0.00)	1.68
Overall	0.00 (0.00, 0.01)	100.00
Q=5758.96, p=0.00, I2=99%		

Triplicated SANa. Two arising from RCA, the third arising from LCX

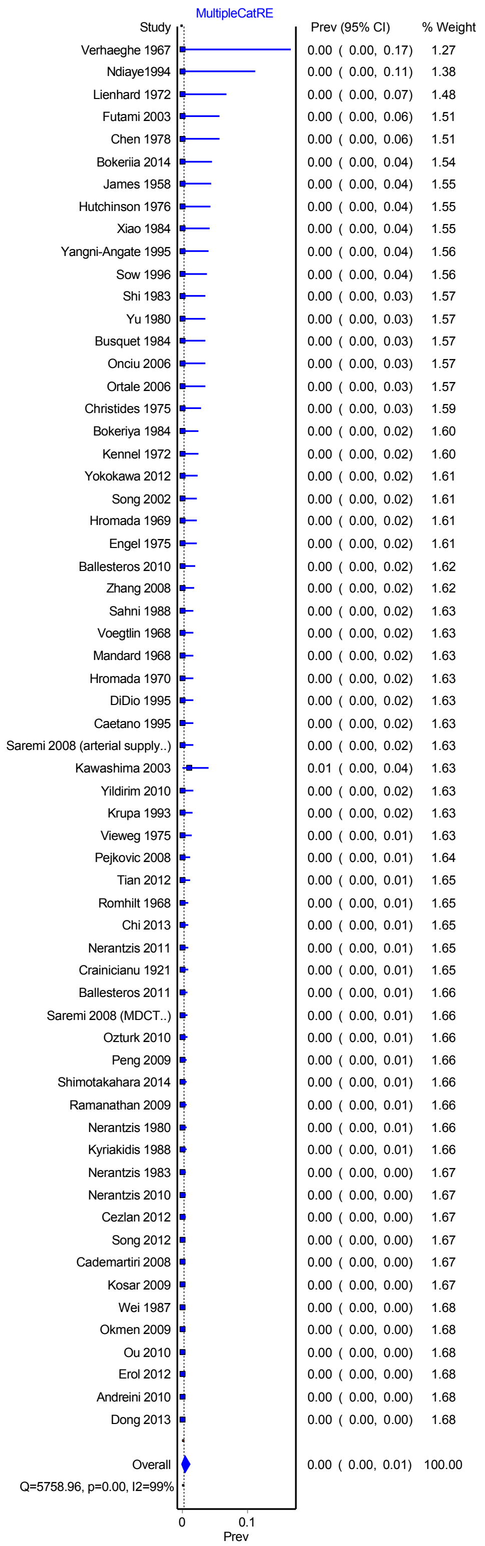

Triplicated SANa. Two arising from LCX, the third arising from RCA

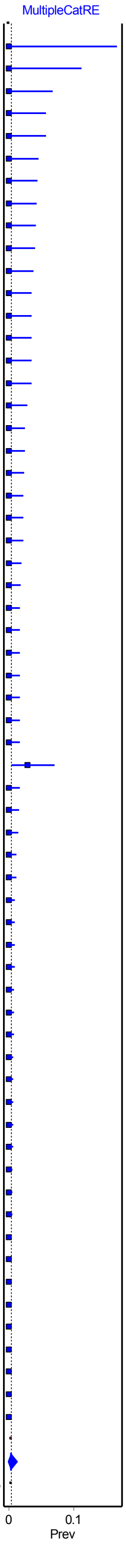

Study	Prev (95% CI)	% Weight
Verhaeghe 1967	0.00 (0.00, 0.17)	1.27
Ndiaye1994	0.00 (0.00, 0.11)	1.38
Lienhard 1972	0.00 (0.00, 0.07)	1.48
Futami 2003	0.00 (0.00, 0.06)	1.51
Chen 1978	0.00 (0.00, 0.06)	1.51
Bokeriia 2014	0.00 (0.00, 0.04)	1.54
James 1958	0.00 (0.00, 0.04)	1.55
Hutchinson 1976	0.00 (0.00, 0.04)	1.55
Xiao 1984	0.00 (0.00, 0.04)	1.55
Yangni-Angate 1995	0.00 (0.00, 0.04)	1.56
Sow 1996	0.00 (0.00, 0.04)	1.56
Shi 1983	0.00 (0.00, 0.03)	1.57
Yu 1980	0.00 (0.00, 0.03)	1.57
Busquet 1984	0.00 (0.00, 0.03)	1.57
Onciu 2006	0.00 (0.00, 0.03)	1.57
Ortale 2006	0.00 (0.00, 0.03)	1.57
Christides 1975	0.00 (0.00, 0.03)	1.59
Bokeriya 1984	0.00 (0.00, 0.02)	1.60
Kennel 1972	0.00 (0.00, 0.02)	1.60
Yokokawa 2012	0.00 (0.00, 0.02)	1.61
Song 2002	0.00 (0.00, 0.02)	1.61
Hromada 1969	0.00 (0.00, 0.02)	1.61
Engel 1975	0.00 (0.00, 0.02)	1.61
Ballesteros 2010	0.00 (0.00, 0.02)	1.62
Zhang 2008	0.00 (0.00, 0.02)	1.62
Sahni 1988	0.00 (0.00, 0.02)	1.63
Voegtlin 1968	0.00 (0.00, 0.02)	1.63
Mandard 1968	0.00 (0.00, 0.02)	1.63
Hromada 1970	0.00 (0.00, 0.02)	1.63
DiDio 1995	0.00 (0.00, 0.02)	1.63
Caetano 1995	0.00 (0.00, 0.02)	1.63
Saremi 2008 (arterial supply..)	0.00 (0.00, 0.02)	1.63
Kawashima 2003	0.03 (0.00, 0.07)	1.63
Yildirim 2010	0.00 (0.00, 0.02)	1.63
Krupa 1993	0.00 (0.00, 0.02)	1.63
Vieweg 1975	0.00 (0.00, 0.01)	1.63
Pejkovic 2008	0.00 (0.00, 0.01)	1.64
Tian 2012	0.00 (0.00, 0.01)	1.65
Romhilt 1968	0.00 (0.00, 0.01)	1.65
Chi 2013	0.00 (0.00, 0.01)	1.65
Nerantzis 2011	0.00 (0.00, 0.01)	1.65
Crainicianu 1921	0.00 (0.00, 0.01)	1.65
Ballesteros 2011	0.00 (0.00, 0.01)	1.66
Saremi 2008 (MDCT..)	0.00 (0.00, 0.01)	1.66
Ozturk 2010	0.00 (0.00, 0.01)	1.66
Peng 2009	0.00 (0.00, 0.01)	1.66
Shimotakahara 2014	0.00 (0.00, 0.01)	1.66
Ramanathan 2009	0.00 (0.00, 0.01)	1.66
Nerantzis 1980	0.00 (0.00, 0.01)	1.66
Kyriakidis 1988	0.00 (0.00, 0.01)	1.66
Nerantzis 1983	0.00 (0.00, 0.00)	1.67
Nerantzis 2010	0.00 (0.00, 0.00)	1.67
Cezlan 2012	0.00 (0.00, 0.00)	1.67
Song 2012	0.00 (0.00, 0.00)	1.67
Cademartiri 2008	0.00 (0.00, 0.00)	1.67
Kosar 2009	0.00 (0.00, 0.00)	1.67
Wei 1987	0.00 (0.00, 0.00)	1.68
Okmen 2009	0.00 (0.00, 0.00)	1.68
Ou 2010	0.00 (0.00, 0.00)	1.68
Erol 2012	0.00 (0.00, 0.00)	1.68
Andreini 2010	0.00 (0.00, 0.00)	1.68
Dong 2013	0.00 (0.00, 0.00)	1.68
Overall	0.00 (0.00, 0.01)	100.00

Q=5758.96, p=0.00, I2=99%

Triplicated SANa. Two arising from RCA, the third arising from LCA

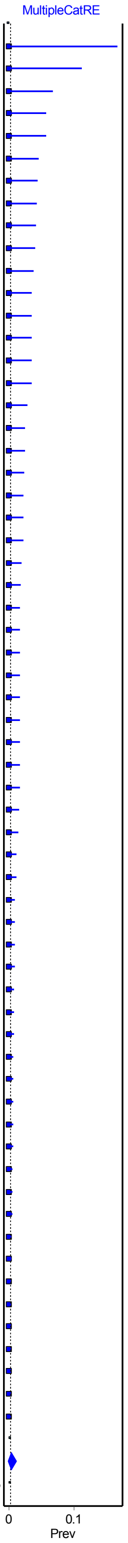

Study	Prev (95% CI)	% Weight
Verhaeghe 1967	0.00 (0.00, 0.17)	1.27
Ndiaye1994	0.00 (0.00, 0.11)	1.38
Lienhard 1972	0.00 (0.00, 0.07)	1.48
Futami 2003	0.00 (0.00, 0.06)	1.51
Chen 1978	0.00 (0.00, 0.06)	1.51
Bokeriia 2014	0.00 (0.00, 0.04)	1.54
James 1958	0.00 (0.00, 0.04)	1.55
Hutchinson 1976	0.00 (0.00, 0.04)	1.55
Xiao 1984	0.00 (0.00, 0.04)	1.55
Yangni-Angate 1995	0.00 (0.00, 0.04)	1.56
Sow 1996	0.00 (0.00, 0.04)	1.56
Shi 1983	0.00 (0.00, 0.03)	1.57
Yu 1980	0.00 (0.00, 0.03)	1.57
Busquet 1984	0.00 (0.00, 0.03)	1.57
Onciu 2006	0.00 (0.00, 0.03)	1.57
Ortale 2006	0.00 (0.00, 0.03)	1.57
Christides 1975	0.00 (0.00, 0.03)	1.59
Bokeriya 1984	0.00 (0.00, 0.02)	1.60
Kennel 1972	0.00 (0.00, 0.02)	1.60
Yokokawa 2012	0.00 (0.00, 0.02)	1.61
Song 2002	0.00 (0.00, 0.02)	1.61
Hromada 1969	0.00 (0.00, 0.02)	1.61
Engel 1975	0.00 (0.00, 0.02)	1.61
Ballesteros 2010	0.00 (0.00, 0.02)	1.62
Zhang 2008	0.00 (0.00, 0.02)	1.62
Sahni 1988	0.00 (0.00, 0.02)	1.63
Voegtlin 1968	0.00 (0.00, 0.02)	1.63
Mandard 1968	0.00 (0.00, 0.02)	1.63
Hromada 1970	0.00 (0.00, 0.02)	1.63
DiDio 1995	0.00 (0.00, 0.02)	1.63
Caetano 1995	0.00 (0.00, 0.02)	1.63
Saremi 2008 (arterial supply..)	0.00 (0.00, 0.02)	1.63
Kawashima 2003	0.00 (0.00, 0.02)	1.63
Yildirim 2010	0.00 (0.00, 0.02)	1.63
Krupa 1993	0.00 (0.00, 0.02)	1.63
Vieweg 1975	0.00 (0.00, 0.01)	1.63
Pejkovic 2008	0.00 (0.00, 0.01)	1.64
Tian 2012	0.00 (0.00, 0.01)	1.65
Romhilt 1968	0.00 (0.00, 0.01)	1.65
Chi 2013	0.00 (0.00, 0.01)	1.65
Nerantzis 2011	0.00 (0.00, 0.01)	1.65
Crainicianu 1921	0.00 (0.00, 0.01)	1.65
Ballesteros 2011	0.00 (0.00, 0.01)	1.66
Saremi 2008 (MDCT..)	0.00 (0.00, 0.01)	1.66
Ozturk 2010	0.00 (0.00, 0.01)	1.66
Peng 2009	0.00 (0.00, 0.01)	1.66
Shimotakahara 2014	0.00 (0.00, 0.01)	1.66
Ramanathan 2009	0.00 (0.00, 0.01)	1.66
Nerantzis 1980	0.00 (0.00, 0.01)	1.66
Kyriakidis 1988	0.00 (0.00, 0.01)	1.66
Nerantzis 1983	0.00 (0.00, 0.00)	1.67
Nerantzis 2010	0.00 (0.00, 0.00)	1.67
Cezlan 2012	0.00 (0.00, 0.00)	1.67
Song 2012	0.00 (0.00, 0.00)	1.67
Cademartiri 2008	0.00 (0.00, 0.00)	1.67
Kosar 2009	0.00 (0.00, 0.00)	1.67
Wei 1987	0.00 (0.00, 0.00)	1.68
Okmen 2009	0.00 (0.00, 0.00)	1.68
Ou 2010	0.00 (0.00, 0.00)	1.68
Erol 2012	0.00 (0.00, 0.00)	1.68
Andreini 2010	0.00 (0.00, 0.00)	1.68
Dong 2013	0.00 (0.00, 0.00)	1.68
Overall	0.00 (0.00, 0.01)	100.00

Q=5758.96, p=0.00, I2=99%

Triplicated SANa. Two arising from RCA, the third arising from bronchial artery

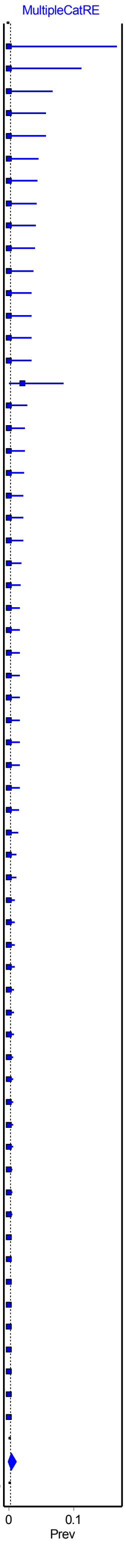

Study	Prev (95% CI)	% Weight
Verhaeghe 1967	0.00 (0.00, 0.17)	1.27
Ndiaye1994	0.00 (0.00, 0.11)	1.38
Lienhard 1972	0.00 (0.00, 0.07)	1.48
Futami 2003	0.00 (0.00, 0.06)	1.51
Chen 1978	0.00 (0.00, 0.06)	1.51
Bokeriia 2014	0.00 (0.00, 0.04)	1.54
James 1958	0.00 (0.00, 0.04)	1.55
Hutchinson 1976	0.00 (0.00, 0.04)	1.55
Xiao 1984	0.00 (0.00, 0.04)	1.55
Yangni-Angate 1995	0.00 (0.00, 0.04)	1.56
Sow 1996	0.00 (0.00, 0.04)	1.56
Shi 1983	0.00 (0.00, 0.03)	1.57
Yu 1980	0.00 (0.00, 0.03)	1.57
Busquet 1984	0.00 (0.00, 0.03)	1.57
Onciu 2006	0.00 (0.00, 0.03)	1.57
Ortale 2006	0.02 (0.00, 0.08)	1.57
Christides 1975	0.00 (0.00, 0.03)	1.59
Bokeriya 1984	0.00 (0.00, 0.02)	1.60
Kennel 1972	0.00 (0.00, 0.02)	1.60
Yokokawa 2012	0.00 (0.00, 0.02)	1.61
Song 2002	0.00 (0.00, 0.02)	1.61
Hromada 1969	0.00 (0.00, 0.02)	1.61
Engel 1975	0.00 (0.00, 0.02)	1.61
Ballesteros 2010	0.00 (0.00, 0.02)	1.62
Zhang 2008	0.00 (0.00, 0.02)	1.62
Sahni 1988	0.00 (0.00, 0.02)	1.63
Voegtlin 1968	0.00 (0.00, 0.02)	1.63
Mandard 1968	0.00 (0.00, 0.02)	1.63
Hromada 1970	0.00 (0.00, 0.02)	1.63
DiDio 1995	0.00 (0.00, 0.02)	1.63
Caetano 1995	0.00 (0.00, 0.02)	1.63
Saremi 2008 (arterial supply..)	0.00 (0.00, 0.02)	1.63
Kawashima 2003	0.00 (0.00, 0.02)	1.63
Yildirim 2010	0.00 (0.00, 0.02)	1.63
Krupa 1993	0.00 (0.00, 0.02)	1.63
Vieweg 1975	0.00 (0.00, 0.01)	1.63
Pejkovic 2008	0.00 (0.00, 0.01)	1.64
Tian 2012	0.00 (0.00, 0.01)	1.65
Romhilt 1968	0.00 (0.00, 0.01)	1.65
Chi 2013	0.00 (0.00, 0.01)	1.65
Nerantzis 2011	0.00 (0.00, 0.01)	1.65
Crainicianu 1921	0.00 (0.00, 0.01)	1.65
Ballesteros 2011	0.00 (0.00, 0.01)	1.66
Saremi 2008 (MDCT..)	0.00 (0.00, 0.01)	1.66
Ozturk 2010	0.00 (0.00, 0.01)	1.66
Peng 2009	0.00 (0.00, 0.01)	1.66
Shimotakahara 2014	0.00 (0.00, 0.01)	1.66
Ramanathan 2009	0.00 (0.00, 0.01)	1.66
Nerantzis 1980	0.00 (0.00, 0.01)	1.66
Kyriakidis 1988	0.00 (0.00, 0.01)	1.66
Nerantzis 1983	0.00 (0.00, 0.00)	1.67
Nerantzis 2010	0.00 (0.00, 0.00)	1.67
Cezlan 2012	0.00 (0.00, 0.00)	1.67
Song 2012	0.00 (0.00, 0.00)	1.67
Cademartiri 2008	0.00 (0.00, 0.00)	1.67
Kosar 2009	0.00 (0.00, 0.00)	1.67
Wei 1987	0.00 (0.00, 0.00)	1.68
Okmen 2009	0.00 (0.00, 0.00)	1.68
Ou 2010	0.00 (0.00, 0.00)	1.68
Erol 2012	0.00 (0.00, 0.00)	1.68
Andreini 2010	0.00 (0.00, 0.00)	1.68
Dong 2013	0.00 (0.00, 0.00)	1.68
Overall	0.00 (0.00, 0.01)	100.00

Q=5758.96, p=0.00, I2=99%

Triplicated SANa. Two arising from bronchial artery, the third arising from RCA

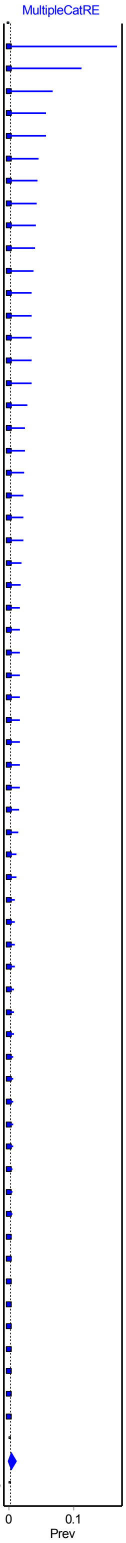

Study	Prev (95% CI)	% Weight
Verhaeghe 1967	0.00 (0.00, 0.17)	1.27
Ndiaye1994	0.00 (0.00, 0.11)	1.38
Lienhard 1972	0.00 (0.00, 0.07)	1.48
Futami 2003	0.00 (0.00, 0.06)	1.51
Chen 1978	0.00 (0.00, 0.06)	1.51
Bokeriia 2014	0.00 (0.00, 0.04)	1.54
James 1958	0.00 (0.00, 0.04)	1.55
Hutchinson 1976	0.00 (0.00, 0.04)	1.55
Xiao 1984	0.00 (0.00, 0.04)	1.55
Yangni-Angate 1995	0.00 (0.00, 0.04)	1.56
Sow 1996	0.00 (0.00, 0.04)	1.56
Shi 1983	0.00 (0.00, 0.03)	1.57
Yu 1980	0.00 (0.00, 0.03)	1.57
Busquet 1984	0.00 (0.00, 0.03)	1.57
Onciu 2006	0.00 (0.00, 0.03)	1.57
Ortale 2006	0.00 (0.00, 0.03)	1.57
Christides 1975	0.00 (0.00, 0.03)	1.59
Bokeriya 1984	0.00 (0.00, 0.02)	1.60
Kennel 1972	0.00 (0.00, 0.02)	1.60
Yokokawa 2012	0.00 (0.00, 0.02)	1.61
Song 2002	0.00 (0.00, 0.02)	1.61
Hromada 1969	0.00 (0.00, 0.02)	1.61
Engel 1975	0.00 (0.00, 0.02)	1.61
Ballesteros 2010	0.00 (0.00, 0.02)	1.62
Zhang 2008	0.00 (0.00, 0.02)	1.62
Sahni 1988	0.00 (0.00, 0.02)	1.63
Voegtlin 1968	0.00 (0.00, 0.02)	1.63
Mandard 1968	0.00 (0.00, 0.02)	1.63
Hromada 1970	0.00 (0.00, 0.02)	1.63
DiDio 1995	0.00 (0.00, 0.02)	1.63
Caetano 1995	0.00 (0.00, 0.02)	1.63
Saremi 2008 (arterial supply..)	0.00 (0.00, 0.02)	1.63
Kawashima 2003	0.00 (0.00, 0.02)	1.63
Yildirim 2010	0.00 (0.00, 0.02)	1.63
Krupa 1993	0.00 (0.00, 0.02)	1.63
Vieweg 1975	0.00 (0.00, 0.01)	1.63
Pejkovic 2008	0.00 (0.00, 0.01)	1.64
Tian 2012	0.00 (0.00, 0.01)	1.65
Romhilt 1968	0.00 (0.00, 0.01)	1.65
Chi 2013	0.00 (0.00, 0.01)	1.65
Nerantzis 2011	0.00 (0.00, 0.01)	1.65
Crainicianu 1921	0.00 (0.00, 0.01)	1.65
Ballesteros 2011	0.00 (0.00, 0.01)	1.66
Saremi 2008 (MDCT..)	0.00 (0.00, 0.01)	1.66
Ozturk 2010	0.00 (0.00, 0.01)	1.66
Peng 2009	0.00 (0.00, 0.01)	1.66
Shimotakahara 2014	0.00 (0.00, 0.01)	1.66
Ramanathan 2009	0.00 (0.00, 0.01)	1.66
Nerantzis 1980	0.00 (0.00, 0.01)	1.66
Kyriakidis 1988	0.00 (0.00, 0.01)	1.66
Nerantzis 1983	0.00 (0.00, 0.00)	1.67
Nerantzis 2010	0.00 (0.00, 0.00)	1.67
Cezlan 2012	0.00 (0.00, 0.00)	1.67
Song 2012	0.00 (0.00, 0.00)	1.67
Cademartiri 2008	0.00 (0.00, 0.00)	1.67
Kosar 2009	0.00 (0.00, 0.00)	1.67
Wei 1987	0.00 (0.00, 0.00)	1.68
Okmen 2009	0.00 (0.00, 0.00)	1.68
Ou 2010	0.00 (0.00, 0.00)	1.68
Erol 2012	0.00 (0.00, 0.00)	1.68
Andreini 2010	0.00 (0.00, 0.00)	1.68
Dong 2013	0.00 (0.00, 0.00)	1.68
Overall	0.00 (0.00, 0.01)	100.00

Q=5758.96, p=0.00, I2=99%

SANa arising from right coronary sinus

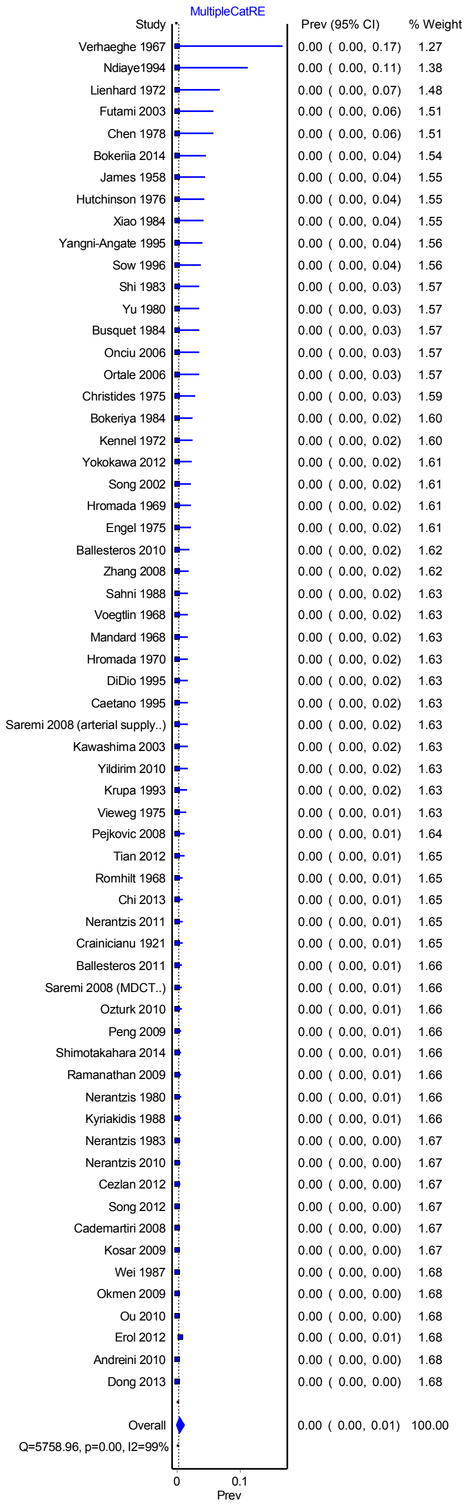

SANa arising from left coronary sinus

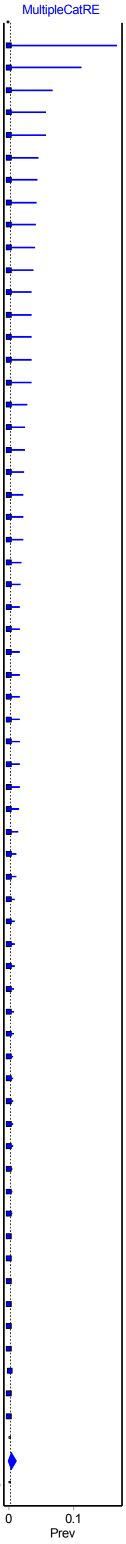

Study	Prev (95% CI)	% Weight
Verhaeghe 1967	0.00 (0.00, 0.17)	1.27
Ndiaye1994	0.00 (0.00, 0.11)	1.38
Lienhard 1972	0.00 (0.00, 0.07)	1.48
Futami 2003	0.00 (0.00, 0.06)	1.51
Chen 1978	0.00 (0.00, 0.06)	1.51
Bokeriia 2014	0.00 (0.00, 0.04)	1.54
James 1958	0.00 (0.00, 0.04)	1.55
Hutchinson 1976	0.00 (0.00, 0.04)	1.55
Xiao 1984	0.00 (0.00, 0.04)	1.55
Yangni-Angate 1995	0.00 (0.00, 0.04)	1.56
Sow 1996	0.00 (0.00, 0.04)	1.56
Shi 1983	0.00 (0.00, 0.03)	1.57
Yu 1980	0.00 (0.00, 0.03)	1.57
Busquet 1984	0.00 (0.00, 0.03)	1.57
Onciu 2006	0.00 (0.00, 0.03)	1.57
Ortale 2006	0.00 (0.00, 0.03)	1.57
Christides 1975	0.00 (0.00, 0.03)	1.59
Bokeriya 1984	0.00 (0.00, 0.02)	1.60
Kennel 1972	0.00 (0.00, 0.02)	1.60
Yokokawa 2012	0.00 (0.00, 0.02)	1.61
Song 2002	0.00 (0.00, 0.02)	1.61
Hromada 1969	0.00 (0.00, 0.02)	1.61
Engel 1975	0.00 (0.00, 0.02)	1.61
Ballesteros 2010	0.00 (0.00, 0.02)	1.62
Zhang 2008	0.00 (0.00, 0.02)	1.62
Sahni 1988	0.00 (0.00, 0.02)	1.63
Voegtlin 1968	0.00 (0.00, 0.02)	1.63
Mandard 1968	0.00 (0.00, 0.02)	1.63
Hromada 1970	0.00 (0.00, 0.02)	1.63
DiDio 1995	0.00 (0.00, 0.02)	1.63
Caetano 1995	0.00 (0.00, 0.02)	1.63
Saremi 2008 (arterial supply..)	0.00 (0.00, 0.02)	1.63
Kawashima 2003	0.00 (0.00, 0.02)	1.63
Yildirim 2010	0.00 (0.00, 0.02)	1.63
Krupa 1993	0.00 (0.00, 0.02)	1.63
Vieweg 1975	0.00 (0.00, 0.01)	1.63
Pejkovic 2008	0.00 (0.00, 0.01)	1.64
Tian 2012	0.00 (0.00, 0.01)	1.65
Romhilt 1968	0.00 (0.00, 0.01)	1.65
Chi 2013	0.00 (0.00, 0.01)	1.65
Nerantzis 2011	0.00 (0.00, 0.01)	1.65
Crainicianu 1921	0.00 (0.00, 0.01)	1.65
Ballesteros 2011	0.00 (0.00, 0.01)	1.66
Saremi 2008 (MDCT..)	0.00 (0.00, 0.01)	1.66
Ozturk 2010	0.00 (0.00, 0.01)	1.66
Peng 2009	0.00 (0.00, 0.01)	1.66
Shimotakahara 2014	0.00 (0.00, 0.01)	1.66
Ramanathan 2009	0.00 (0.00, 0.01)	1.66
Nerantzis 1980	0.00 (0.00, 0.01)	1.66
Kyriakidis 1988	0.00 (0.00, 0.01)	1.66
Nerantzis 1983	0.00 (0.00, 0.00)	1.67
Nerantzis 2010	0.00 (0.00, 0.00)	1.67
Cezlan 2012	0.00 (0.00, 0.00)	1.67
Song 2012	0.00 (0.00, 0.00)	1.67
Cademartiri 2008	0.00 (0.00, 0.00)	1.67
Kosar 2009	0.00 (0.00, 0.00)	1.67
Wei 1987	0.00 (0.00, 0.00)	1.68
Okmen 2009	0.00 (0.00, 0.00)	1.68
Ou 2010	0.00 (0.00, 0.00)	1.68
Erol 2012	0.00 (0.00, 0.00)	1.68
Andreini 2010	0.00 (0.00, 0.00)	1.68
Dong 2013	0.00 (0.00, 0.00)	1.68
Overall	0.00 (0.00, 0.01)	100.00

Q=5758.96, p=0.00, I2=99%
