# Supplementary figures and images for: Anatomical Variations in the Sinoatrial Nodal Artery: A Meta-Analysis and Clinical Considerations
Source: PLoS One. 2016 Feb 5;11(2):e0148331. doi: 10.1371/journal.pone.0148331 (PMC4743947; doi:10.1371/journal.pone.0148331)

## S-Shaped sinoatrial nodal artery

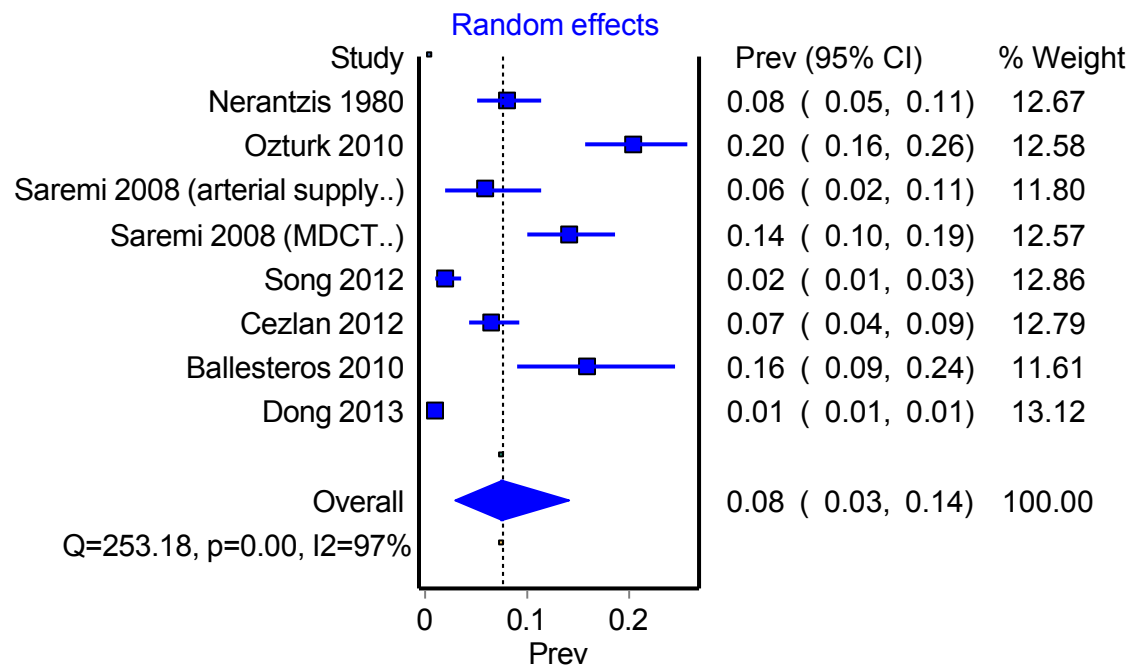

Supplement: S4 Fig — (PDF) [file pone.0148331.s005.pdf]
